# Theoretical Calculations for Highly Selective Direct Heteroarylation Polymerization: New Nitrile-Substituted Dithienyl-Diketopyrrolopyrrole-Based Polymers

**DOI:** 10.3390/molecules23092324

**Published:** 2018-09-12

**Authors:** Thomas Bura, Serge Beaupré, Marc-André Légaré, Olzhas A. Ibraikulov, Nicolas Leclerc, Mario Leclerc

**Affiliations:** 1 Canada Research Chair on Electroactive and Photoactive Polymers, Department of Chemistry, Université Laval, Quebec City, QC G1V 0A6, Canada; thomas.bura.1@ulaval.ca (T.B.); serge.beaupre.1@ulaval.ca (S.B.); 2 Institut für Anorganische Chemie, Julius-Maximilians Universität Würzburg, Am Hubland, 97074 Würzburg, Germany; marcandrelegare@gmail.com; 3 Laboratoire ICube, DESSP, Université de Strasbourg, CNRS, 23 rue du Loess, 67037 Strasbourg, France; ibraikulov@unistra.fr; 4 Institut de Chimie et Procédés pour l’Énergie, l’Environnement et la Santé, ICPEES, Université de Strasbourg, CNRS, 67087 Strasbourg, France; leclercn@unistra.fr

**Keywords:** DHAP, selectivity, theoretical calculations, conjugated polymers, organic electronics

## Abstract

Direct Heteroarylation Polymerization (DHAP) is becoming a valuable alternative to classical polymerization methods being used to synthesize π-conjugated polymers for organic electronics applications. In previous work, we showed that theoretical calculations on activation energy (E_a_) of the C–H bonds were helpful to rationalize and predict the selectivity of the DHAP. For readers’ convenience, we have gathered in this work all our previous theoretical calculations on E_a_ and performed new ones. Those theoretical calculations cover now most of the widely utilized electron-rich and electron-poor moieties studied in organic electronics like dithienyl-diketopyrrolopyrrole (DT-DPP) derivatives. Theoretical calculations reported herein show strong modulation of the E_a_ of C–H bond on DT-DPP when a bromine atom or strong electron withdrawing groups (such as fluorine or nitrile) are added to the thienyl moiety. Based on those theoretical calculations, new cyanated dithienyl-diketopyrrolopyrrole (CNDT-DPP) monomers and copolymers were prepared by DHAP and their electro-optical properties were compared with their non-fluorinated and fluorinated analogues.

## 1. Introduction

Organic solar cells (OSCs) and organic field-effect transistors (OFETs) based on π-conjugated polymers are widely studied to create efficient, lightweight and flexible electronic devices using inexpensive and environmentally friendly printing techniques [1,2,3,4,5,6]. With power conversion efficiency (PCE) exceeding 10% [7,8,9,10,11] and OFETs with hole mobility up to 20 cm^2^ V^−1^ s^−1^ [12] and electron mobility as high as 7.0 cm^2^ V^−1^ s^−1^ [13], conjugated polymers have now reached performances suitable for commercial applications. This is especially true if these materials can be prepared from a low-cost, eco-friendly, reliable and scalable polymerization method such as direct heteroarylation polymerization (DHAP). Indeed, DHAP is becoming a valuable alternative to classical polymerization methods used in organic electronics. Despite its recent development, this new polymerization method has already been the subject of several comprehensive reviews and book chapters [14,15,16,17,18,19,20,21,22]. Significant work is underway to understand and limit some unwanted reactions such as β-branching and homocoupling when several C–H bonds can be activated during the polymerization. New catalytic systems [23,24,25], β-protecting group with no harm on the backbone coplanarity [26] and theoretical calculations on activation energy of C–H bonds [27] are among the actual tools to better understand, limit and eliminate such unwanted reactions. 

Among all materials studied in organic electronics, 2,5-dihydropyrrolo[3,4-c]pyrrole-1,4-dione (DPP)-based polymers are on the short list of materials that show high performances in both OSCs (PCE up to 9.4%) [28] and OFETs (hole mobility up to 10.5 cm^2^ V^−1^ s^−1^) [29]. Since the first report on the synthesis of 3,6-diphenyl-DPP by Farnum et al. in 1974 [30], DPP-based copolymers have been extensively studied and reviewed [31,32,33,34]. The flanking aromatic substituents (five- or six-membered-fused or unfused heterocyclic rings) strongly modulate the electro-optical properties. Flanking thiophenes have minimal steric effects on the DPP core and lead to co-planar dithienyl-DPP building blocks (DT-DPP). Recently, we have reported that fluorination of dithienyl-DPP-based monomers (fDT-DPP) is a valuable strategy to obtain well-defined, defect-free DPP-based copolymers by DHAP with enhanced performances in OSCs and OFETs compared to their non-fluorinated analogues [26]. Indeed, a PCE of 7.5% was obtained for fDT-DPP/carbazole copolymer which was twice that of non-fluorinated counterpart. This PCE value is among the highest values reported so far for OSCs based on conjugated polymers obtained by DHAP [35,36]. 

Besides fluorination, other electron-withdrawing substituent such as nitrile groups have also been used in conjugated polymers to tailor their electronic properties [37,38,39,40,41,42,43,44,45,46,47]. As reported in the literature, cyanation of the conjugated backbone is an efficient strategy to modulate the electro-optical properties of conjugated polymers and in some cases, it can lead to materials with better performances compared to their fluorinated analogues [48]. The nitrile group is a strong electron-withdrawing group that strongly stabilizes the LUMO energy level (without raising the HOMO level) which contributes to reduce the band gap of the resulting copolymers [43,46]. Nitrile groups have also been shown to induce strong dipole moments and promote balanced ambipolar charge transport [49,50]. The main drawback of the cyanation on the conjugated backbone is the lower solubility compared to their non-cyanated analogues which lead to low molecular weight materials with limited processability [42]. 

In this work, we have summarized all of our theoretical calculations performed on activation energy of the C–H bonds of most of the widely used electron-rich and electron-poor moieties studied in organic electronics. In previous work, we found that the E_a_ values were helpful to rationalize and predict regio-selectivity of the DHAP when multiple C–H bonds can be activated during the polymerization [26,27]. Based on new theoretical calculations, we then synthesized cyanated dithienyl-Diketopyrrolopyrrole (CNDT-DPP) derivatives suitable for DHAP. CNDT-DPP comonomers were then polymerized with either dithienyl-DPP (DT-DPP) or 2,7-dibromocarbazole derivatives and the electro-optical properties of the resulting CNDT-DPP copolymers were compared with their non-cyanated and fluorinated analogues. 

## 2. Results and Discussion

### 2.1. Theoretical Calculations

**Figure 1:**
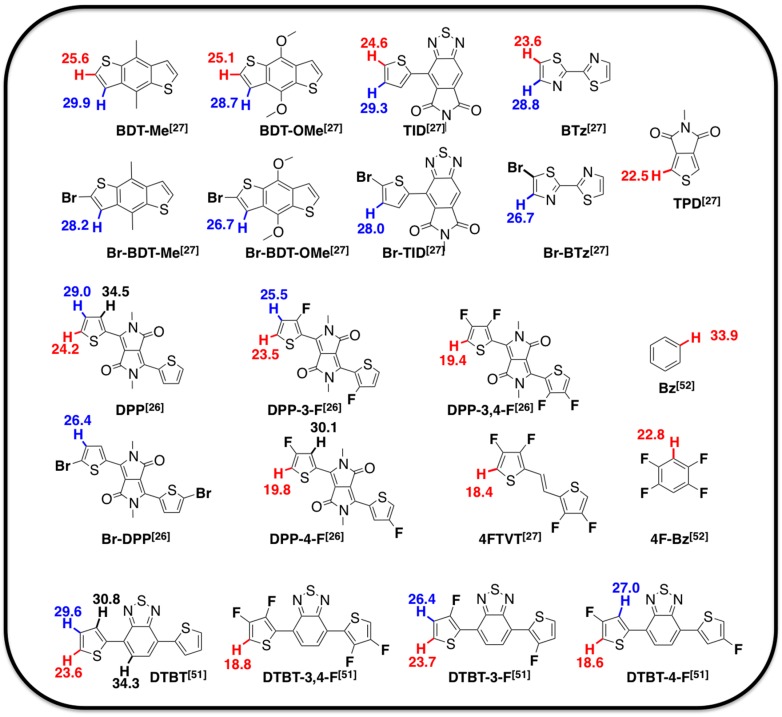
Gibbs free energy of the CMD transition state associated to the transition state for the C–H bond cleavage (previous work: [26,27,51,52]).

**Figure 2:**
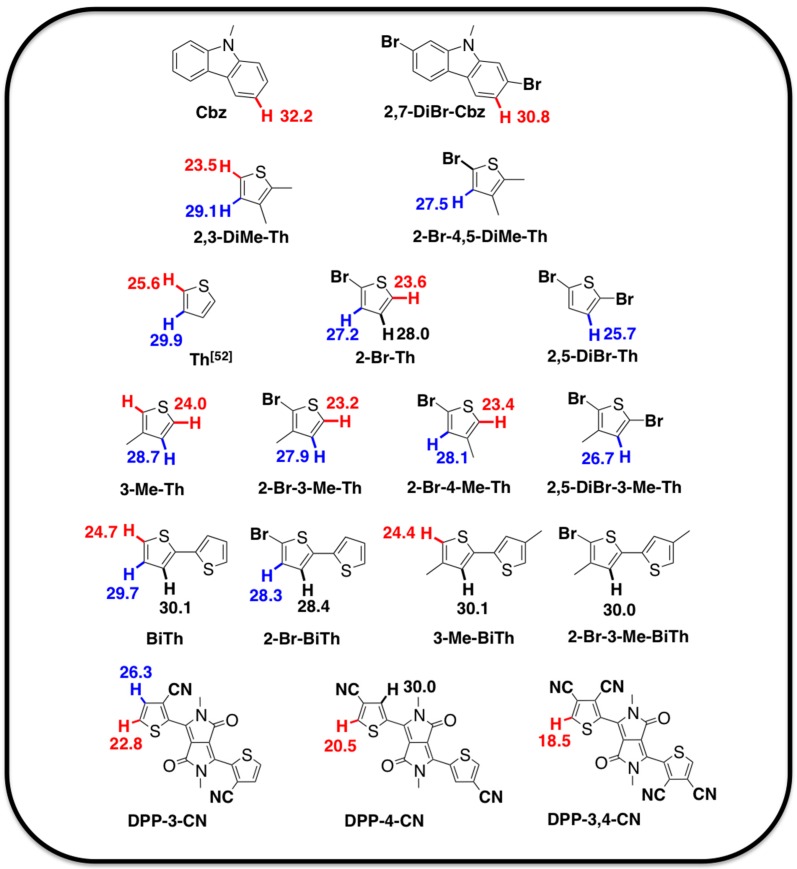
Gibbs free energy of the CMD transition state associated to the transition state for the C–H bond cleavage (this work [52]).

DFT calculations at the B3LYP/TZVP (DZVP for palladium) level have been carried out to evaluate the activation barrier of the C-H bond activation of the Concerted-Metalation Deprotonation (CMD) pathway during DHAP. The model palladium catalyst (PMe_3_)Pd(Ph)(CH_3_COO-) was used as a platform to calculate the Gibbs free energy of the CMD transition state associated to the activation of the different C–H bonds of each substrate. The activation energy (E_a_, Figure 1 and Figure 2) refers to the Gibbs free energy of the CMD transition state referenced to the substrate and model catalyst (ΔG^‡^_298_) as reported in previous studies [26,27,51,52]. These calculations are relevant to α versus β selectivity of DHAP for possible β-defects but do not say anything about potential α-α homocouplings. From those theoretical calculations, it is also possible to estimate (using Arrhenius’s law) a selectivity ratio between H_α_ and H_β_ at the temperature of polymerization [26]. More importantly, these calculations reveal the importance of the pairing of the comonomers used in DHAP. 

Indeed, the data indicate that the presence of bromine atoms (Figure 1 and Figure 2) decreases the energy of activation (E_a_) of the adjacent C–H bonds, allowing undesirable β-defects for some brominated aromatic units. For instance, if we utilized the DHAP with Br-BDT-Me or Br-BDT-OMe and DPP (Figure 1); the difference in the activation energy (ΔE_a_) between H_α_ and H_β_ for the DPP moiety is found to be 4.8 kcal∙mol^−1^ (24.2 vs. 29.0 kcal∙mol^−1^, respectively), which lead to a ratio of about 250:1 favoring H_α_ (using Arrhenius’s law). On the other hand, the E_a_ of the H_β_ of the Br-BDT-Me is 28.2 kcal∙mol^−1^ whereas the E_a_ for H_β_ on the Br-BDT-OMe is only 26.7 kcal∙mol^−1^. Using these values, the ΔE_a_ between H_α_ of DPP (24.2 kcal∙mol^−1^) and the H_β_ of the Br-BDT-OMe (26.7 kcal∙mol^−1^) and Br-BDT-Me (28.2 kcal∙mol^−1^) are respectively of 2.5 and 4.0 kcal∙mol^−1^, giving respective selectivity of about 25:1 and 160:1 at 125 °C. Although the 1.5 kcal∙mol^−1^ difference might look small, it indicates that the activation of the H_β_ of the Br-BDT-OMe may occur about one order of magnitude faster than the one of the H_β_ of the Br-BDT-Me resulting in more β-defects, while the polymer using Br-BDT-Me will better behave in term of selectivity. 

These calculations indicate that some brominated monomers are not intrinsically inadequate but their utilization is strongly dependent upon the nature and reactivity of the respective comonomer. This has been proven by the work of Li et al. [53]. Indeed, well-defined copolymer was obtained from the copolymerization of Br-DPP with bithiazole (BTz) while poor performances were obtained for the same copolymer synthesized using Br-BTz and DPP. For the first case, the ΔE_a_ between the H_α_ (23.6 kcal∙mol^−1^) and H_β_ (28.8 kcal∙mol^−1^) of BTz is 5.2 kcal∙mol^−1^ which means that the activation of the H_α_ position is highly selective (ratio 1152:1). Meanwhile, a selectivity of about 35:1 is observed when considering the ΔE_a_ value of 2.8 kcal∙mol^−1^ between H_β_ of the Br-DPP (26.4 kcal∙mol^−1^) and H_α_ of the BTz (23.6 kcal∙mol^−1^). On the other hand, for the Br-BTz/DPP system, the ΔE_a_ between H_β_ of the Br-BTz (26.7 kcal∙mol^−1^) and the H_α_ of the DPP (24.2 kcal∙mol^−1^) is 2.5 kcal∙mol^−1^ (ratio 23:1) and should lead to less-defined structure. 

### 2.2. Justification for Cyano-Dithienyl-DPP (CNDT-DPP) 

**Scheme 1:**
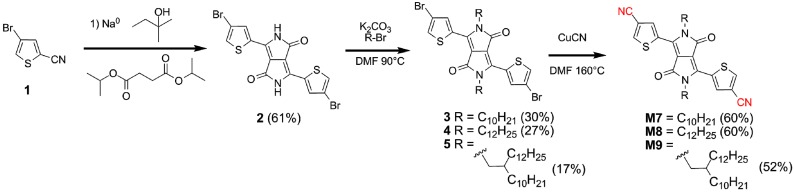
Synthetic Pathway for the Synthesis of CN-DPP Comonomers **M7**, **M8** and **M9**.

**Scheme 2:**
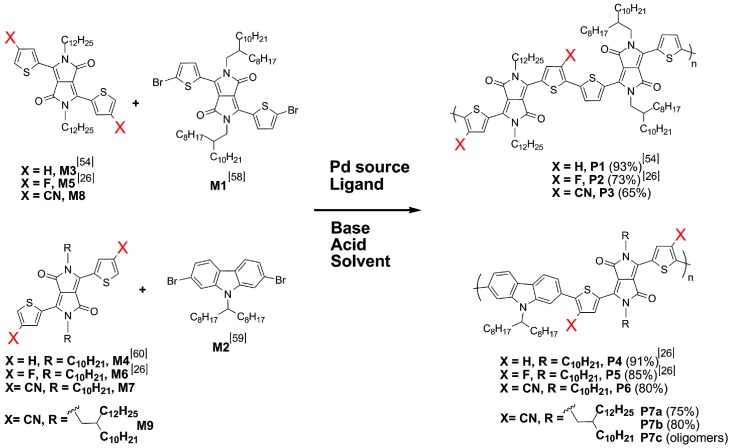
Monomers and Polymers Investigated in this Work. [26,54,55,56,57].

Lately, we have shown that the fluorination of flanking thiophene (at the 3- or 4-position) influences the electro-optical properties and the coplanarity of the DPP-based copolymers and to obtain a coplanar fluorinated DPP, the fluorine atom has to be installed at the 4-position of thiophene ring [26,51]. A fluorine atom at the 4-position on flanking thiophene strongly decreases the activation energy of the adjacent C–H bond (from 24.2 kcal∙mol^−1^ to 19.8 kcal∙mol^−1^) for DPP moiety (Figure 1). We then performed a similar study by replacing the fluorine atom by a nitrile group, a strong electron-withdrawing moiety. As shown in Figure 2, the nitrile groups have also a strong influence on the adjacent C–H bonds. Indeed, we found a ΔE_a_ of 3.7 kcal∙mol^−1^ for H_α_ of DPP-4-CN (20.5 kcal∙mol^−1^) vs DPP (24.2 kcal∙mol^−1^) and 4.5 kcal∙mol^−1^ for Hγ of DPP-4-CN (30.0 kcal∙mol^−1^) vs. DPP (34.5 kcal∙mol^−1^). Although, the activation energy of Hγ is decreased by 4.5 kcal∙mol^−1^ compared to DPP, a selectivity of about 500,000/1 in favor of H_α_ over Hγ was calculated using Arrhenius’s law. This means that polymerization reaction should mainly occur at the α-position and lead to well-defined materials. The same trend was observed for DPP-3-CN vs. DPP-3-F and DPP-3,4-CN vs. DPP-3,4-F. Keeping in mind the need for coplanar copolymers, only DPP-4-CN was synthesized (Scheme 1) and copolymerized by DHAP (Scheme 2).

### 2.3. Synthesis of Monomers

A pseudo Stobbe condensation between diisopropyl succinic ester and commercially available 4-bromothiophene-2-carbonitrile (**1**) in the presence of sodium alkoxide led to 3,6-(4-bromo-thiophen-2-yl)pyrrolo[3,4-c]-pyrrole-1,4-dione (**2**) in moderate yield (61%, Scheme 1). In the next step, a typical alkylation reaction of the DPP core was performed using either 1-bromodecane, 1-bromododecane or 1-bromo-2-decyltetradecane and allowed us to obtain corresponding alkylated DPP **3**–**5** in low yields (17–30%). Finally, the target monomers **M7**–**M9** (see Supplementary Materials) were obtained in good yield by cyanation of the respective brominated DPP through a Rosenmund-von Braun cyano-dehalogenation reaction with CuCN in hot anhydrous DMF as described in the literature [49]. For comparison purposes, **M7**–**M9** were obtained in three steps with an overall yield of 10% while **M5** and **M6** (fDT-DPP analogues) [26] were obtained in seven steps with an overall yield of 8%. 

### 2.4. Synthesis and Characterization of Polymers

**P3**, **P6** and **P7** were prepared by direct (hetero)arylation polymerization (DHAP) following methodologies previously reported in the literature (Scheme 2) [26]. Cyanated dithienyl-DPP (CNDT-DPP) pseudo-homopolymer **P3** was synthesized for future OFETs applications while CNDT-DPP/carbazole copolymers **P6**, **P7** were designed to be used as an electron-donor in OSCs. According to theoretical calculations, the reaction between Br-DPP **M1** and DPP-4-CN **M8** (Figure 1 and Figure 2) should proceed smoothly without β-branching on the DPP-4-CN or on the Br-DPP moieties. Indeed, the β-position is protected by the nitrile group on the DPP-4-CN and the H_α_ has an E_a_ of 20.5 kcal∙mol^−1^ while the E_a_ of H_β_ on the Br-DPP is 26.4 kcal∙mol^−1^. The ΔE_a_ between H_β_ of Br-DPP (26.4 kcal∙mol^−1^) and the H_α_ of DPP-4-CN (20.5 kcal∙mol^−1^) is 5.9 kcal∙mol^−1^ which lead to an estimated selectivity of 1800:1 in favor of the H_α_ of the DPP-4-CN. The same reasoning should also apply for **P6** involving the reaction between **M2** (Scheme 2) and **M7** (Figure 2). The β-position on DPP-4-CN is protected by the nitrile group and the ΔE_a_ between H_α_ of DPP-4-CN (20.5 kcal mol^−1^) and the H_α_ of Br-Cbz (30.8 kcal∙mol^−1^) is 10.3 kcal∙mol^−1^ which lead to an estimated selectivity of 687,000:1 in favor of the H_α_ of DPP-4-CN. It is worth nothing that **P3** and **P6** were synthesized using the same catalytic conditions as those used for their fluorinated analogues [26]. Unlike fDT-DPP based copolymers **P2** [26], the polymerization reaction of **P3** was stopped rapidly (10 min) due to gelation of the reaction mixture. 

As shown in Table 1, the polymerization time for **P3** (10 min) and **P6** (40 min) were shorter compared to the polymerization times of DT-DPP analogues (1200 min for **P1** and 960 min for **P4**). As for fDT-DPP comonomers, the shorter polymerization times compared to those observed for the non-fluorinated are due to strong electron-withdrawing effect of the cyano group on the α C–H bond. Moreover, the polymerization times for **P6** vs **P5** were similar which might mean that the reactivity of **M7** and **M6** are similar, which is in good agreement with the E_a_ of DPP-4-CN (20.5 kcal∙mol^−1^) vs. E_a_ of DPP-4-F (19.8 kcal∙mol^−1^). Unlike **P2,** we were unable to determine the molecular weights of **P3** due to strong aggregation and limited solubility, even in TCB at 135 °C. On the other hand, the molecular weight of **P6** (soluble fraction) was lower compared to **P4** and **P5**. The short polymerization time (40 min), due to rapid gelation of the reaction mixture, led to a material with low molecular weight. In the hope of promoting the solubility of CNDT-DPP/Carbazole copolymers, long and branched alkyl side chains were installed on the CNDT-DPP moiety (see **M9** in Scheme 1). **P7a** was obtained by DHAP from **M2** and **M9** using the same catalytic system used for the synthesis of **P6** but in more dilute conditions in superheated tetrahydrofuran (C = 0.085 mol∙L^−1^ for **P7a** vs. C = 0.1 mol∙L^−1^ for **P6**). Despite a longer polymerization time before gelation of the reaction mixture happened for **P7a** (240 min), a lower molecular weight material was obtained. **P7b** was then synthesized using the same polymerization conditions used for **P6** (C = 0.1 mol∙L^−1^ in THF)**.** As for **P6**, gelation of the polymerization mixture was observed after only 40 min. Despite higher $\bar{M_{n}}$ (35 kg mol^−1^) the degree of polymerization of **P7b** and **P6** are the same (DP = 24). Finally, **P7c** was synthesized using chlorobenzene instead of THF (C = 0.1 mol∙L^−1^) but the polymerization did not work and only oligomers were recovered. **P6** was then used for comparison purposes with **P4** and **P5** since these polymers have the same side chains installed on the DPP moiety.

Thermal properties were evaluated by thermogravimetric analysis (TGA) and differential scanning calorimetry (DSC). All polymers exhibit good thermal stability with 5% weight loss at temperature higher than 400 °C (see Table 1). Differential scanning calorimetry measurements did not reveal any thermal transition. NMR spectroscopy was useless to identify any defect within the conjugated backbone for **P3** and **P6** due to broad and featureless signals. 

**Figure 3:**
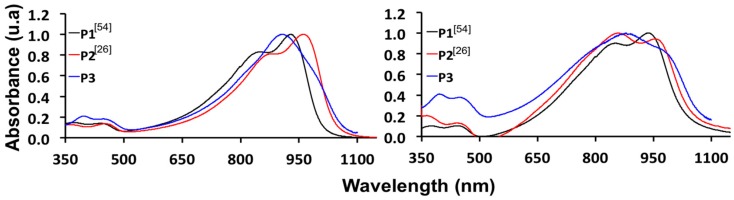
UV-Visible absorption spectra of **P1**–**P3** in solution in CHCl_3_ (**left**) and in the solid state (**right**) [26,54].

**Figure 4:**
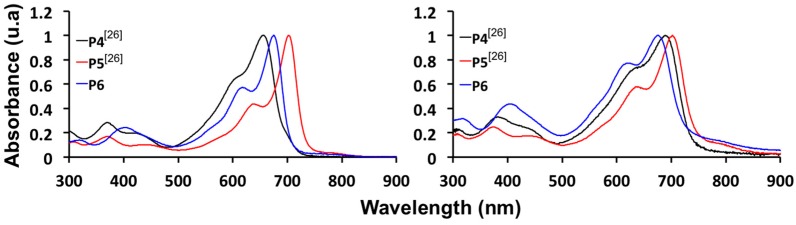
UV-Visible absorption spectra of **P4**–**P6** in solution in CHCl_3_ (**left**) and in the solid state (**right**) [26].

**Figure 5:**
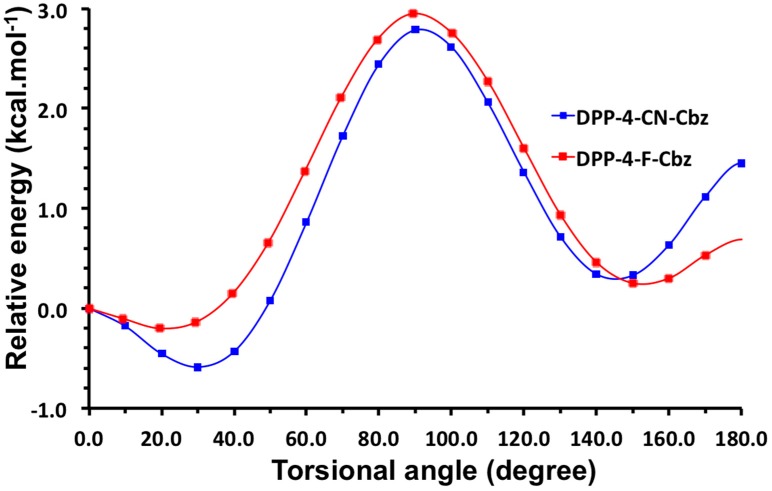
Ground-state energy surfaces of CBz-DPP-4-F and CBz-DPP-4-CN dimers.

The UV-Vis-NIR absorption spectroscopy (solution and film) was performed to gauge the effect of substituents at 4-position on flanking thiophene DPP core on the optical properties and the results are summarized in Table 2. The absorption spectra of **P1**–**P6** are shown in Figure 3 and Figure 4. In dilute chloroform solution, a bathochromic shift of the maximum of absorption (19 nm) was observed for **P6** (674 nm) compared to **P4** (655 nm). In the same condition, a hypsochromic shift of 28 nm was observed for the maximum of absorption of **P6** (674 nm) compared to the maximum of absorption of **P5** (702 nm). Beside the fact that $\bar{M_{n}}$ of **P5** (125 kg mol^−1^) is much higher than **P6** (25 kg mol^−1^), the optical band gaps were found to be almost identical (1.68 eV for **P6** and 1.65 eV for **P5**). The hypsochromic shift observed for **P6** is due to a conformational twisting of the conjugated backbone due to the larger size of the nitrile group compared to fluorine which was confirmed by conformational analyses performed on the repeating unit of **P5** and **P6** (Figure 5). The calculations suggest that **P6** is more twisted than **P5** (30.6° vs. 24.0°), which should reduce the effective length of conjugation of the polymer. 

**Figure 6:**
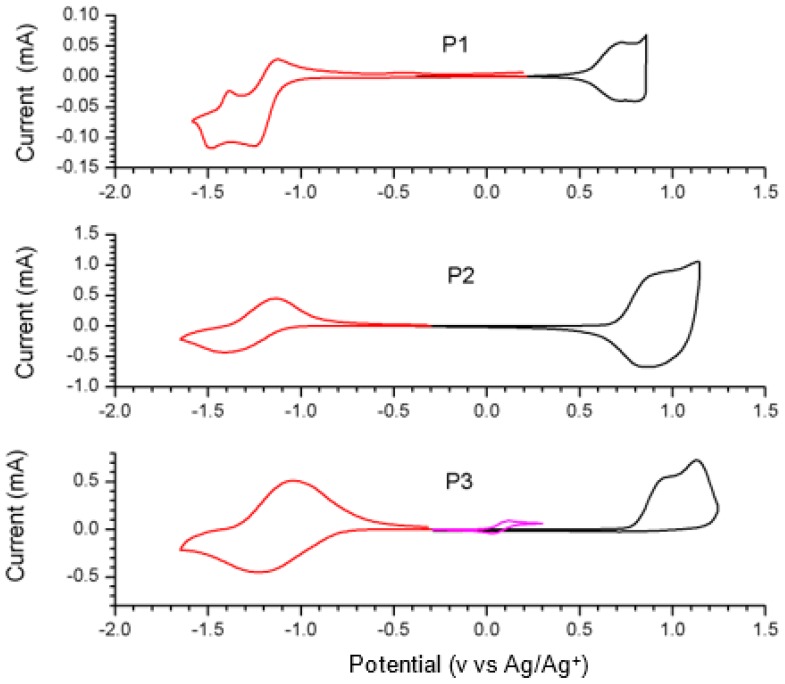
Cyclic voltammetry of **P1** [54], **P2 [26]** and **P3** (In magenta, Fc/Fc+).

Electrochemical measurements such as cyclic voltammetry are a widely used method in organic electronics to estimate the HOMO and LUMO energy levels analyses. In order to be able to compare the values reported in literature, it is of the utmost importance that the measurements are performed under the same conditions (electrolyte, working electrode, reference electrode (non-aqueous is highly desirable), scanning speed, determination of the onset oxidation and reduction, and calibration against Ferrocene). Comparisons between datasets can be therefore misleading. Lately, we found that some HOMO/LUMO values reported might have been over-estimated. This issue has been fixed and the HOMO/LUMO values reported in Table 2 reflects those changes. As example, we have previously reported for **P1** a HOMO energy level of −5.45 eV and a LUMO energy level of −4.14 eV [54]. After corrections the HOMO and LUMO energy levels are now −5.13 eV and −3.82 eV, which is in good agreement with DPP-based polymers reported in literature [33]. The same corrections have been done for **P2** and **P5** previously reported in literature [26]. For **P1**–**P3**, a stabilization of the HOMO energy level was observed upon fluorination and cyanation of the DPP moiety (Figure 6).

**Figure 7:**
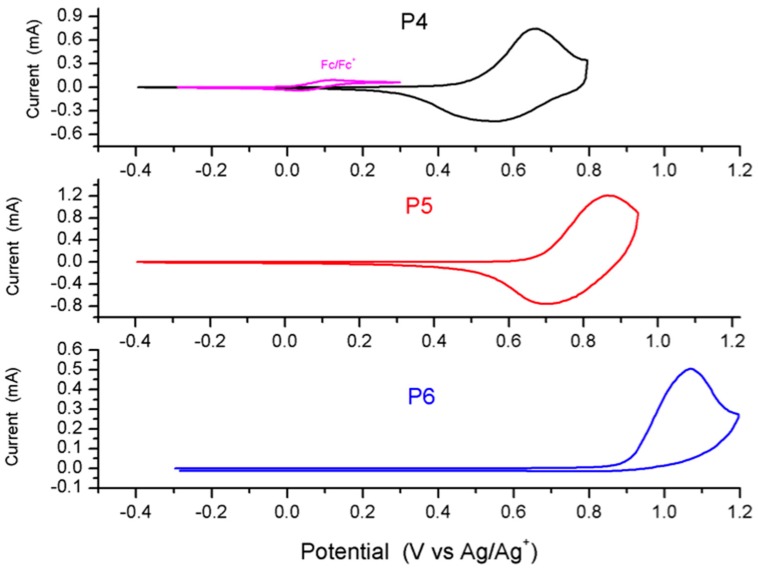
Cyclic voltammetry of P4 [26], P5 [26] and P6.

**Figure 8:**
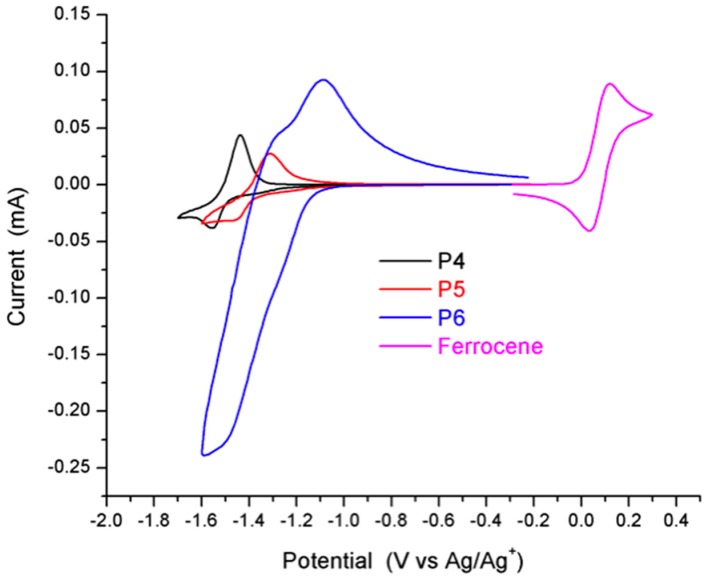
Cyclic voltammetry of **P4** [26], **P5** [26] and **P6** (Reduction only).

Indeed, the HOMO level of **P2** (−5.35 eV) was stabilized by 0.22 eV while the HOMO level of **P3** (−5.44 eV) was stabilized by 0.31 eV compared to HOMO level of **P1** (−5.13 eV). The same trend was observed for **P4**–**P6** (Figure 7). The stabilization of the HOMO level due to cyanation of DPP moiety was even larger for **P6** (0.43 eV). These data are in good agreement with the trend observed for the cyanation of conjugated polymers. It is worth nothing that deeper HOMO energy levels might lead to a higher open circuit voltage (V_oc_) in OSCs. On the other hand, the LUMO energy levels were also determined from cyclic voltammetry even though reduction measurements are more sensitive to water and oxygen. While the estimated LUMO energy levels obtained by cyclic voltammetry for **P4**–**P6** (Figure 8) led to $E_{g}^{cv}$ similar to $E_{g}^{opt}$, it was not the case for the **P1**–**P3** (Figure 6). Indeed, even if reversible reduction processes were observed for each polymer, the band gap (calculated from the reduction and the oxidation onsets) led to $E_{g}^{cv}$ values that did not match the optical band gap calculated from solid state UV-Vis absorption spectra (Table 2). We found a ΔE_g_ between $E_{g}^{cv}$ and $E_{g}^{opt}$ of 0.14 eV, 0.51 eV and 0.32 eV for **P1**, **P2** and **P3** respectively. Even if stabilization of the LUMO energy levels was observed upon cyanation of the DT-DPP moiety (−3.82 eV to −3.96 eV, Figure 6), it appears that the reduction processes observed for **P1** to **P3** might not correspond to the extraction of electron from the conjugated skeleton but may come from the reduction of other functional group found on the DPP moiety. As reported in literature, the discrepancy between the electrochemical and optical band gaps may also be ascribed to the strong exciton binding energy in conjugated polymers. This binding energy can be as high as 0.4–1.0 eV [58,59,60]. We then calculated the LUMO energy levels using the optical band gap and the HOMO energy levels (Table 2). Going from **P1** to **P3**, we noticed a steady stabilization of the LUMO energy levels −3.96 eV, −4.20 eV and −4.28 eV. These deep LUMO energy levels make **P2** and **P3** good candidates for *n*-type materials for OFETs or good electron-acceptor in all polymer solar cells (PSCs). **P3** and **P6** are currently tested in OFETs and PSCs and those results will be reported in a forthcoming paper. 

## 3. Materials and Methods

^1^H and ^13^C-NMR spectra were recorded on a Varian AS400 or Agilent DD2 500 MHz apparatus (Santa Clara, CA, USA) in deuterated solvents. Chemical shifts were reported as δ values (ppm) relative to the residual protic solvent (see Supplementary Materials). Infrared Spectra were obtained using a Specac spectrometer using golden gate single reflection attenuated total reflection system (Pleasantville, NY, USA) fitted with a diamond crystal. The infrared spectra were recorded in solid state with a Nicolet Magna 550 Fourier transform spectrometer (Madison, WI, USA) equipped with a narrow band mercury-cadmiun-telluride (MCT) detector and a germanium-coated KBr beam splitter. A total of 64 interferograms were acquired, co-added and Fourier transformed to give a spectral resolution of 4 cm^−^^1^ in the spectral range of 4000–800 cm^−^^1^. High resolution mass spectra were recorded with a time-of-flight spectrometer in flow infusion analysis on an Agilent Technologies 1200 series system (Santa Clara, CA, USA). Ions were generated by a Kr UV lamp in an APPI source with toluene/anisole mixture as dopant when required. Spectra ranging from 100 to 3200 *m*/*z* were recorded with typical resolution of 5000 to 15,000 and typical precision of ±1 ppm or less. The mass analyzer was calibrated with standard solutions provided by the manufacturer and covering the spectral range described above. The number-average ($\bar{M_{n}}$) and weight-average ($\bar{M_{W}}$) molecular weights were determined by size exclusion chromatography (SEC) using a Varian Polymer Laboratories PL-GPC 120 (Church Stretton, Shropshire, United Kingdom). The column set consists of 2 PL gel Mixed C (300 × 7.5 mm) columns and a PL gel Mixed C guard column. The flow rate was fixed at 1 mL/min using 1,2,4-trichlorobenzene (TCB) (with 0.0125% BHT *w/v*) as eluent. The temperature of the system was set to 110 °C. All the samples were prepared at concentrations of nominally 1.0 mg/mL in TCB. Dissolution was performed using a Varian Polymer Laboratories PL-AS RT autosampler. The sample vials were held at 110 °C with shaking for 1 h for complete dissolution. The solutions were filtered through a 2 mm porous stainless steel filter used with the 0.40 μm glass filter into a 2 mL chromatography vial. The calibration method used to generate the reported data was the classical polystyrene method using polystyrene narrow standards Easi-Vials PS-M from Varian Polymer Laboratories which were dissolved in TCB. UV-Vis absorption spectra were taken using a Thermo Scientific Genesys 10S spectrophotometer (Madison, WI, USA) using 1 cm path-length quartz cells. For solid state measurements, polymer solution was spun-cast on glass plates. Optical bandgaps were calculated from the onset of the absorption band. Thermogravimetric analysis (TGA) measurements were carried out with a Mettler Toledo TGA SDTA 851e apparatus (Colombus, OH, USA) at a heating rate of 10 °C/min under a nitrogen atmosphere. The temperature of degradation (Td) corresponds to a 5% weight loss. Differential scanning calorimetry (DSC) analysis was performed on a Mettler Toledo DSC823e apparatus (Colombus, OH, USA) calibrated with ultrapure indium at a scanning rate of 10 °C/min under a nitrogen flow. Cyclic voltammograms were recorded on a Solartron 1287 potentiostat (Oak Ridge, TN, USA) using platinum wires as working electrode and counter electrode at a scan rate of 50 mV/s. The reference electrode was Ag/Ag^+^ (0.01 M AgNO_3_ in acetonitrile), and the electrolyte was a solution of 0.1 M tetrabutylammonium tetrafluoroborate (Bu_4_NBF_4_) in dry acetonitrile. In these conditions, the half-wave oxidation potential of ferrocene was 0.091 V versus Ag/Ag + ($E_{Ferrocene\;vs.\;Ag/Ag^{+}}^{1/2}$). The HOMO and LUMO energy levels were determined from the oxidation and reduction onsets (E′) (where the current differs from the baseline) using the following equations:

$$E_{\mathrm{HOMO}}=-\left[\left(E_{ox\;vs.\;Ag/Ag^{+}}^{\prime }\right)+4.8-\left(E_{Ferrocene\;vs.\;Ag/Ag^{+}}^{1/2}\right)\right]$$

$$E_{\mathrm{LUMO}}=-\left[\left(E_{red\;vs.\;Ag/Ag^{+}}^{\prime }\right)+4.8-\left(E_{Ferrocene\;vs.\;Ag/Ag^{+}}^{1/2}\right)\right]$$

### 3.1. Materials

4-Bromothiophene-2-carbonitrile (**1**) was purchased from Combiblocks (San Diego, CA, USA)*.* **P1 [54]**, **P2 [26]**, **P4** [26], **P5** [26], 3,6-bis(5-bromothiophen-2-yl)-2,5-bis(2-octyl-dodecyl)pyrrolo[3,4-c]pyrrole-1,4)-dione (**M1**) [55], 2,7-dibromo-9-(heptadecan-9-yl)-9H-carbazole (**M2**) [56], 3,6-bis(thiophen-2-yl)-2,5-bis(dodecyl)pyrrolo[3,4-c]pyrrole-1,4-dione (**M3**) [54], 3,6-bis-(thiophen-2-yl)-2,5-bis(decyl)-pyrrolo[3,4-c]pyrrole-1,4-dione(**M4**) [57], 3,6-(4-fluorothiophen-2-yl)-2,5-Bis(dodecyl)pyrrolo[3,4-c]pyrrole-1,4-dione (**M5**) [26], 3,6-(4-fluorothiophen-2-yl)-2,5-Bis(decyl)pyrrolo[3,4-c]pyrrole-1,4-dione (**M6**) [26] and 4-tris(2-cycloheptyloxyphenyl)phosphine (Bura-Phos) [27] were synthesized according to literature.

### 3.2. Synthesis of Monomers and Polymers

#### 3.2.1. Synthesis of 3,6-(4-Bromothiophen-2-yl)pyrrolo[3,4-c]-pyrrole-1,4-dione (**2**)

In a two-neck flask equipped with a condenser and an addition funnel, sodium (3.67 g, 0.159 mol, 1.2 eq.) was added in 2-methyl-2-butanol (250 mL, and the mixture was heated at 105 °C until complete consumption of the sodium. Then, 4-bromo-2-thiophenecarbonitrile (**1**, 25 g, 0.133 mol, 1 eq.) was rapidly added into the mixture, and diisopropyl succinate (12.1 g, 59.8 mmol, 0.45 eq.) was slowly added through the addition funnel. The reaction mixture quickly turned purple. The reaction mixture was stirred overnight at 105 °C. The reaction was cooled at 65 °C and quenched by an addition of a mixture of methanol (150 mL) and acetic acid (70 mL) followed by heating at 90 °C for 30 min. Then, the reaction mixture was allowed to cool to room temperature, filtered on a Buchner, and washed with methanol to give a purple solid (Y = 61%).

#### 3.2.2. Synthesis of 3,6-(4-Bromothiophen-2-yl)-2,5-bis(decyl)-pyrrolo[3,4-c]pyrrole-1,4-dione (**3**)

In a two-neck flask equipped with a condenser and an addition funnel, compound **2** (1.150 g, 2.51 mmol, 1 eq.) and anhydrous potassium carbonate (1.1 g, 7.53 mmol, 3 eq.) were stirred in anhydrous DMF (8 mL). The mixture was heated at 100 °C for 30 min, and then 1-bromodecane (1.7 g, 7.53 mmol, 3 eq.) was slowly added. The reaction mixture was stirred overnight at 100 °C. After cooling to room temperature, the reaction was quenched with a saturated solution of NH_4_Cl and extracted three times with diethyl ether. The combined organic phases were washed with water and brine, dried over MgSO_4_, and concentrated under vacuum. Purification was achieved by column chromatography (silica gel; eluent: chloroform/hexanes 85/15) affording the desired compound as a purple solid (Y = 30%). ^1^H-NMR (500 MHz, CDCl_3_) δ (ppm): 8.81 (s, 2H), 7.52 (s, 2H), 4.02–3.99 (m, 4H), 1.73–1.70 (m, 4H), 1.42–1.26 (m, 28H), 0.90–0.87 (m, 6H). ^13^C-NMR (126 MHz, CDCl_3_) δ (ppm): 169.9, 138.8, 136.7, 130.5, 127.7, 112.3, 108.2, 42.3, 31.9, 29.9, 29.6, 29.5, 29.3, 29.2, 26.8, 22.6, 14.1. m.p. = 126 °C. FTIR ν (cm^−^^1^) = 3094, 2951, 2916, 2850, 1666, 1576, 1407, 883. HRMS (APPI+): *m*/*z* calcd for C_34_H_47_Br_2_N_2_O_2_S_2_: 737.1446; found: 737.1475 [M + H]^+^

#### 3.2.3. Synthesis of 3,6-(4-Bromo-thiophen-2-yl)-2,5-bis(dodecyl)-pyrrolo[3,4-c]pyrrole-1,4-dione (**4**)

Compound **4** was synthesized and purified according to the procedure described for compound **3** using 1-bromododecane instead of 1-bromodecane affording the desired compound as a purple solid (Y = 27%). ^1^H-NMR (500 MHz, CDCl_3_) δ (ppm): 8.81 (s, 2H), 7.52 (s, 2H), 4.02–3.99 (m, 4H), 1.73–1.70 (m, 4H), 1.42–1.26 (m, 36H), 0.90–0.87 (m, 6H). ^13^C-NMR (126 MHz, CDCl_3_) δ (ppm): 169.9, 138.8, 136.7, 130.5, 127.7, 112.3, 108.2, 42.3, 31.9, 29.9, 29.6, 29.56, 29.52, 29.3, 29.2, 26.8, 22.6, 14.1. m.p. =132 °C. FTIR ν (cm^−1^) = 3102, 2917, 2850, 1670, 1557, 1412, 876. HRMS (APPI+): *m*/*z* calcd for C_38_H_55_Br_2_N_2_O_2_S_2_: 793.2072; found: 793.2083 [M + H]^+^

#### 3.2.4. Synthesis of 3,6-(4-Bromo-thiophen-2-yl)-2,5-di(2′-decyltetradecyl)-pyrrolo[3,4-c]pyrrole-1,4-dione (**5**)

Compound **5** was synthesized and purified according to the procedure described for compound **3** using 1-bromo-2-decyltetradecane instead of 1-bromodecane affording the desired compound as a purple solid (Y = 17%). ^1^H-NMR (400 MHz, CDCl_3_) δ (ppm): 8.69 (d, *J =* 1.4 Hz, 2H), 7.50 (d, *J =* 1.4 Hz, 2H), 3.95 (d, *J =* 7.7 Hz, 4H), 1.85 (m, 2H), 1.27–1.21 (m, 80H), 0.89–0.85 (m, 12H). ^13^C-NMR (101 MHz, CDCl_3_) δ (ppm): 161.3, 139.2, 136.4, 130.6, 127.5, 112.1, 108.1, 46.2, 30.0, 29.7, 29.6, 29.5, 29.4, 29.3, 29.2, 22.7, 14.1. HRMS (APPI+): *m*/*z* calcd for C_62_H_103_Br_2_N_2_O_2_S_2_: 1129.5828; found: 1129.5838 [M + H]^+^.

#### 3.2.5. Synthesis of 3,6-(4-Cyano-thiophen-2-yl)-2,5-bis(decyl)-pyrrolo[3,4-c]pyrrole-1,4-dione (**M7**)

In a microwave vial containing compound **3** (230 mg, 0.311 mmol 1 eq.) and copper(I)cyanide (223 mg, 2.49 mmol, 8 eq.) was added dry DMF (3 mL, C = 0.75 mol∙L^−1^). The mixture was heated under argon for 24 h at 160 °C. The reaction mixture was allowed to cool down to room temperature and water was added and the crude was extracted three times with diethyl ether. The aqueous phase was extracted three times with diethyl ether. The solvent was removed under reduced pressure and the residue was purified by column chromatography (chloroform/ethyl acetate, 95/5) affording the desired compound as a purple solid (Y = 60%) ^1^H-NMR (500 MHz, CDCl_3_) δ (ppm): 8.99 (s, 2H), 8.17 (s, 2H), 4.03–4.01 (m, 4H), 1.72–1.69 (m, 4H), 1.42–1.26 (m, 28H), 0.89–0.86 (m, 6H). ^13^C-NMR (126 MHz, CDCl_3_) δ (ppm): 160.7, 138.7, 138.3, 135.2, 131.1, 113.9, 112.5, 108.8, 42.3, 31.8, 30.1, 29.5, 29.3, 29,2, 26.8, 22.6, 14.1. m.p. = 216 °C. FTIR ν (cm^−1^) = 3081, 2914, 2846, 2233, 1667, 1559, 883. HRMS (APPI+): *m*/*z* calcd for C_36_H_47_N_4_O_2_S_2_: 631.3140; found: 631.3177 [M + H]^+^.

#### 3.2.6. Synthesis of 3,6-(4-Cyano-thiophen-2-yl)-2,5-bis(dodecyl)-pyrrolo[3,4-c]pyrrole-1,4-dione (**M8**)

**M8** was synthesized from compound **4** and purified according to the procedure described for **M7**. (Y = 60%). ^1^H-NMR (500 MHz, CDCl_3_) δ (ppm): 8.99 (s, 2H), 8.16 (s, 2H), 4.03–4.00 (m, 4H), 1.70–1.68 (m, 4H), 1.40–1.25 (m, 36H), 0.88–0.86 (m, 6H). ^13^C-NMR (126 MHz, CDCl_3_) δ (ppm): 160.8, 138.8, 138.4, 135.3, 131.3, 114.1, 112.7, 109.0, 42.4, 32.0, 30.1, 29.6, 29.4, 29,3, 26.9, 22.8, 14.2. m.p. = 230 °C. FTIR ν (cm^−1^) = 3088, 2919, 2850, 2236, 1674, 1559, 884. HRMS (APPI+): *m*/*z* calcd for C_40_H_55_N_4_O_2_S_2_: 687.3766; found: 687.3775 [M + H]^+^.

#### 3.2.7. Synthesis of 3,6-(4-Cyano-thiophen-2-yl)-2,5-bis(2-decyltetradecyl)-pyrrolo[3,4-c]pyrrole-1,4-dione (**M9**)

**M9** was synthesized from compound **4** and purified according to the procedure described for **M7** and **M8**. (Y = 52%). ^1^H-NMR (400 MHz, CDCl_3_) δ (ppm): 8.91 (d, *J =* 1.2 Hz, 2H), 8.15 (d, *J =* 1.2 Hz, 2H), 3.95 (d, *J* = 7.7 Hz, 4H), 1.78(br, 2H), 1.39–1.09 (m, 80H), 0.96–0.78 (m, 12H). ^13^C-NMR (101 MHz, CDCl_3_) δ (ppm): 161.1, 139.0, 138.1, 135.1, 131.2, 113.9, 112.4, 109.0, 46.3, 31.9, 29.9, 29.7, 29.6, 29.5, 26.2, 22.6, 14.1. HRMS (APPI+): *m*/*z* calcd for C_63_H_103_N_4_O_2_S_2_: 1023.7522; found: 1023.7546 [M + H]^+^.

#### 3.2.8. Synthesis of **P3**

**M1** (0.076 mmol, 1 eq.), **M8** (0.076 mmol, 1 eq.), *trans*-bis(acetato)bis[*o*-(di-*o*-tolyl- phosphino)benzyl]dipalladium (II) (2% mol), tris(2-methoxyphenyl)phosphine (8% mol), Cs_2_CO_3_ (3 eq.) and pivalic acid (1 eq.) were put in a microwave vial with a magnetic stirring bar. The vial was sealed with a cap and then purged with nitrogen to remove the oxygen (3X). Degassed and anhydrous toluene was added (C = 0.2 mol∙L^−1^, 0.4 mL) and the microwave vial was heated at 120 °C using a slow temperature ramp. After heating for 5 min, gelation of the reaction mixture occurred and the reaction was stopped. Chloroform was added to solubilize the mixture (2 mL) and then the mixture was poured in methanol/acidified water (10% HCl; 9:1 ratio), and the solid was recovered by filtration using a 0.45 μm nylon filter. The polymer was washed using a Soxhlet apparatus with acetone, hexanes, then chloroform. The chloroform fraction was reduced to 5–10 mL and then poured in methanol. The polymer was recovered by filtration over a 0.45 μm nylon filter and dried under vacuum (Y = 65%).

#### 3.2.9. Synthesis of **P6**

**M2** (0.12 mmol, 1 eq.), **M7** (0.12 mmol, 1 eq.), Pd(OAc)_2_ (4% mol), tris(2-cyclo-heptyloxyphenyl)phosphine (BuraPhos) (16% mol), Cs_2_CO_3_ (3 eq.) and pivalic acid (1 eq.) were put in a microwave vial with a magnetic stirring bar. The vial was sealed with a cap and then purged with nitrogen to remove the oxygen (3X). Degassed and anhydrous THF was added (C = 0.1 mol∙L^−1^, 1.2 mL) and the reaction was heated with an oil bath pre-heated at 100 °C (reaction under pressure) until gelation of the reaction mixture. After 40 min, gelation of the reaction mixture occurred and the reaction was stopped. The reaction was cooled to 65 °C and then 2 mL of ODCB was added and the mixture was heated at 100 °C for 5 min. The mixture was poured in methanol/acidified water (10% HCl; 9:1 ratio), and the solid was recovered by filtration using a 0.45 μm nylon filter. The polymer was washed using a Soxhlet apparatus with acetone, hexane, and then chlorobenzene. The chlorobenzene fraction was reduced to 5–10 mL and then poured in methanol. The polymer was recovered by filtration over a 0.45 μm nylon filter and dried under vacuum (Y = 80%).

#### 3.2.10. Synthesis of **P7a**

**M9** (0.143 mmol, 1 eq.), **M7** (0.143 mmol, 1 eq.), Pd(OAc)_2_ (4% mol), tris(2-cyclo-heptyloxyphenyl)phosphine (BuraPhos) (16% mol), Cs_2_CO_3_ (3 eq.) and pivalic acid (1 eq.) were put in a microwave vial with a magnetic stirring bar. The vial was sealed with a cap and then purged with nitrogen to remove the oxygen (3X). Degassed and anhydrous THF was added (C = 0.085 mol∙L^−1^, 1.7 mL) and the reaction was heated with an oil bath pre-heated at 100 °C (reaction under pressure). After 4h the reaction was stopped. The reaction was cooled to 65 °C and then 2 mL of ODCB was added and the mixture was heated at 100 °C for 5 min. The mixture was poured in methanol/acidified water (10% HCl; 9:1 ratio), and the solid was recovered by filtration using a 0.45 μm nylon filter. The polymer was washed using a Soxhlet apparatus with acetone and hexanes. The hexanes fraction was reduced to 5–10 mL and then poured in methanol. The polymer was recovered by filtration over a 0.45 μm nylon filter and dried under vacuum (Y = 75%).

#### 3.2.11. Synthesis of **P7b**

**M9** (0.13 mmol, 1 eq.), **M7** (0.13 mmol, 1 eq.), Pd(OAc)_2_ (4% mol), tris(2-cyclo-heptyloxyphenyl)phosphine (BuraPhos) (16% mol), Cs_2_CO_3_ (3 eq.) and pivalic acid (1 eq.) were put in a microwave vial with a magnetic stirring bar. The vial was sealed with a cap and then purged with nitrogen to remove the oxygen (3X). Degassed and anhydrous THF was added (C = 0.15 mol∙L^−1^, 0.9 mL) and the reaction was heated with an oil bath pre-heated at 100 °C (reaction under pressure) until gelation of the reaction mixture. After 40 min, gelation of the reaction mixture occurred and the reaction was stopped. The reaction was cooled to 65 °C and then 2 mL of ODCB was added and the mixture was heated at 100 °C for 5 min. The mixture was poured in methanol/acidified water (10% HCl; 9:1 ratio), and the solid was recovered by filtration using a 0.45 μm nylon filter. The polymer was washed using a Soxhlet apparatus with acetone, hexanes and chloroform. The chloroform fraction was reduced to 5–10 mL and then poured in methanol. The polymer was recovered by filtration over a 0.45 μm nylon filter and dried under vacuum (Y = 80%).

#### 3.2.12. Synthesis of **P7c**

**M9** (0.143 mmol, 1 eq.), **M7** (0.143 mmol, 1 eq.), Pd(OAc)_2_ (4% mol), tris(2-cyclo-heptyloxyphenyl)phosphine (BuraPhos) (16% mol), Cs_2_CO_3_ (3 eq.) and pivalic acid (1 eq.) were put in a microwave vial with a magnetic stirring bar. The vial was sealed with a cap and then purged with nitrogen to remove the oxygen (3X). Degassed and anhydrous Chlorobenzene was added (C = 0.1 mol∙L^−1^, 1.4 mL) and the reaction was heated with an oil bath pre-heated at 130 °C (reaction under pressure). After 24h the reaction was stopped. The reaction was cooled to 65 °C was poured in methanol/acidified water (10% HCl; 9:1 ratio), and the solid was recovered by filtration using a 0.45 μm nylon filter. The polymer was washed using a Soxhlet apparatus using acetone and only oligomers were recovered.

## 4. Conclusions

We have performed theoretical calculations on new electron-rich and electron-poor compounds for applications in organic electronics. These results led to the design and synthesis of new cyanated dithienyl-diketopyrrolopyrrole units (CNDT-DPP) that were suitable for the direct heteroarylation polymerization. Two classes of cyanated dithienyl-DPP-based copolymers (pseudo-homopolymer (**P3**) and push/pull copolymer (**P6**)) have been prepared and characterized. Upon cyanation, a strong stabilization of the LUMO energy level was observed for **P3** and **P6.** Based on their promising electro-optical properties, **P3** is currently tested as *n*-type material in OFETs and as an electron-acceptor for the development of all-polymer solar cells while **P6** is currently tested as an electron-donating material in OSCs. 

## Figures and Tables

**Table 1:** Characterization of **P1**–**P7a**–**c**.

	$\;\bar{M_{n}}\;$	ᴆ	Yield	Reaction Time	T_d_
	kg mol^−1^		%	min	°C
P1 [54]	16	2.4	93	1200	n.a
P2 [26]	22	3.0	73	1200	430
P3	n.a.	n.a.	65	10	420
P4 [26]	44	3.0	91	960	420
P5 [26]	125	3.0	85	30	420
P6	25	3.5	80	40	405
P7a	20	1.7	75	240	405
P7b	35	2.4	80	40	410
P7c	oligomers			-	-

$\bar{M_{n}}$: number-average molecular weight; ᴆ: Dispersity Index.

**Table 2:** Electro-optical properties of **P1**–**P6**.

	HOMO ^a^	LUMO ^a^	$E_{g}^{cv}$	LUMO ^b^	λ_max_		$E_{g}^{opt}$
					Solution	Film	
	eV	eV	eV	eV	nm	nm	eV
P1 [54]	−5.13	−3.82	1.31	−3.96	820/920	846/927	1.17
P2 [26]	−5.35	−3.69	1.66	−4.20	887/962	859/967	1.15
P3	−5.44	−3.96	1.48	−4.28	907	881	1.16
P4 [26]	−5.11	−3.46	1.65	−3.43	622/655	630/689	1.68
P5 [26]	−5.33	−3.61	1.72	−3.68	639/702	639/702	1.65
P6	−5.54	−3.79	1.75	−3.86	621/674	620/676	1.68
P7a	-5.54	-3.79	1.75	−3.86	620/673	621/674	1.73
P7b	−5.54	−3.79	1.75	−3.86	621/674	621/676	1.72

^a^ from CV measurements. ^b^ E_LUMO_ = E_HOMO_ + $E_{g}^{opt}$.

## Supplementary Materials

The following are available online, Figures S1–S13 ^1^H and ^13^C-NMR spectra, Figures S14–S17 Infrared spectra and Figures S18–S20 GPC chromatogram.
