# Supplementary material for: Theoretical Calculations for Highly Selective Direct Heteroarylation Polymerization: New Nitrile-Substituted Dithienyl-Diketopyrrolopyrrole-Based Polymers
Source: Molecules. 2018 Sep 12;23(9):2324. doi: 10.3390/molecules23092324 (PMC6225168; doi:10.3390/molecules23092324)
Supplement: Supplementary file 1 [file molecules-23-02324-s001.pdf]

SUPPLEMENTARY MATERIAL

Theoretical Calculations for Highly Selective Direct Heteroarylation Polymerization: New Nitrile-Substituted Dithienyl-Diketopyrrolopyrrole-Based Polymers

Thomas Bura [1,†], Serge Beaupré [1,†], Marc-André Légaré [2], Olzhas A. Ibraikulov [3], Nicolas Leclerc [4] and Mario Leclerc [1,*]

1 Canada Research Chair on Electroactive and Photoactive Polymers, Department of Chemistry, Université Laval, G1V 0A6 Quebec City, Canada; thomas.bura.1@ulaval.ca (T.B.); serge.beaupre.1@ulaval.ca (S.B.)

2 Institut für Anorganische Chemie, Julius-Maximilians Universität Würzburg, Am Hubland, 97074 Würzburg, Germany; marcandrelegare@gmail.com

3 Laboratoire ICube, DESSP, Université de Strasbourg, CNRS, 23 rue du Loess, 67037 Strasbourg, France; ibraikulov@unistra.fr

4 Institut de Chimie et Procédés pour l'Énergie, l'Environnement et la Santé, ICPEES, Université de Strasbourg, CNRS, 67087 Strasbourg, France; leclercn@unistra.fr

* Correspondence: Mario.Leclerc@chm.ulaval.ca

Figures S1-S13: ^{1}H-and ^{13}C-NMR spectra 2-8

Figures S14-S17: Infrared spectra 9-10

Figures S18-S20: GPC chromatograms for polymers **P6, P7a and P7b** 11-12

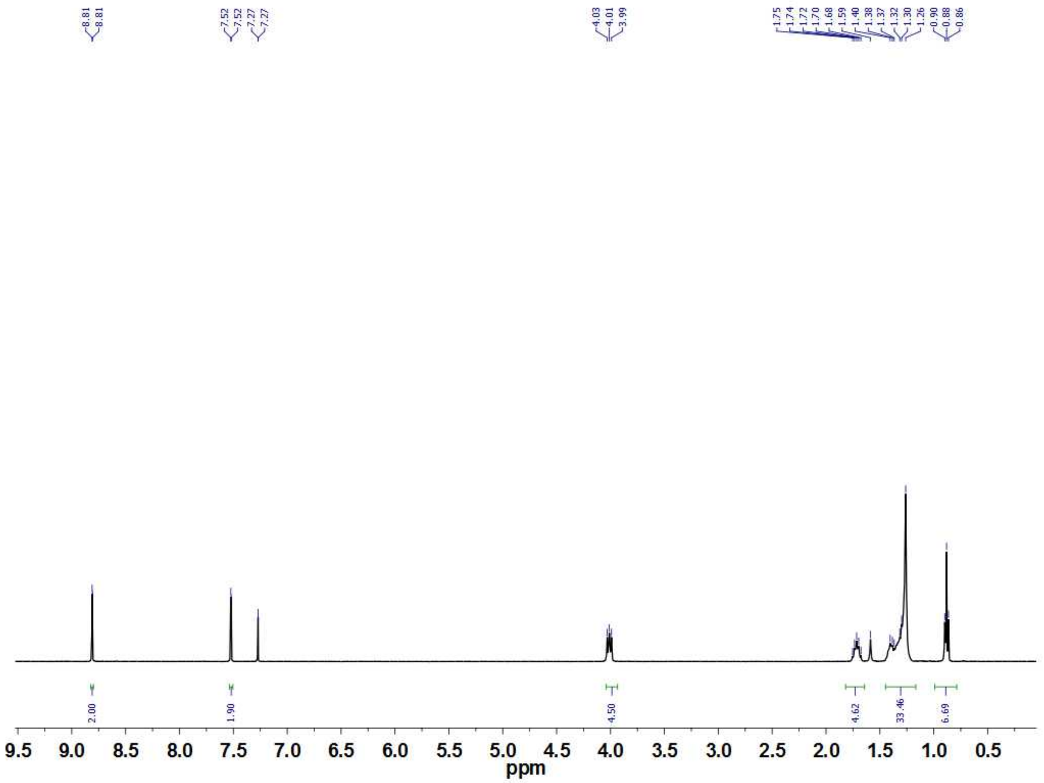

Figure S1 : ^{1}H-NMR spectrum in $CDCl_3$ of compound **3**.

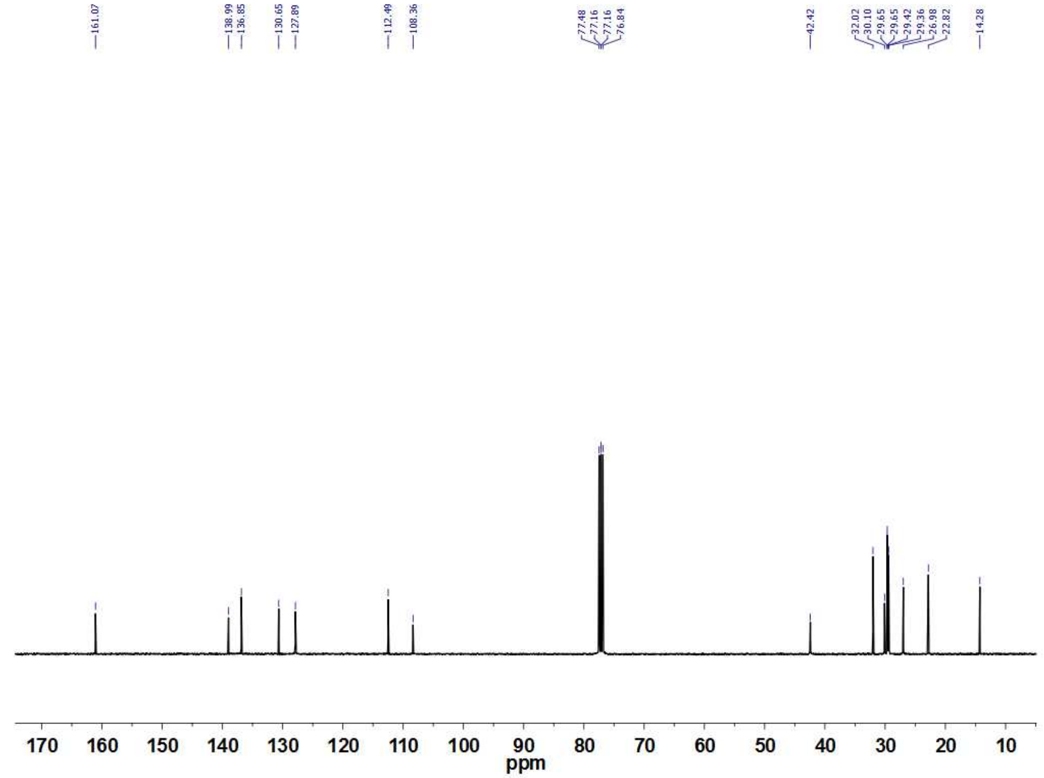

Figure S2 : ^{13}C-NMR spectrum in $CDCl_3$ of compound **3**.

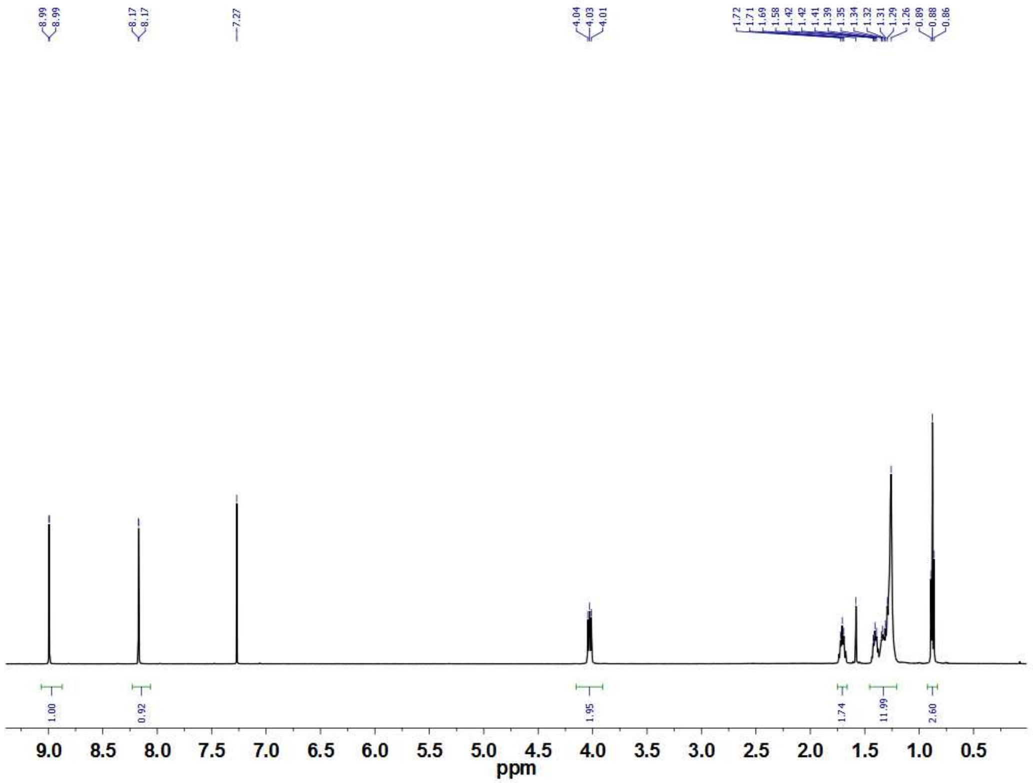

Figure S3 : ^{1}H-NMR spectrum in $CDCl_3$ of compound **M7**.

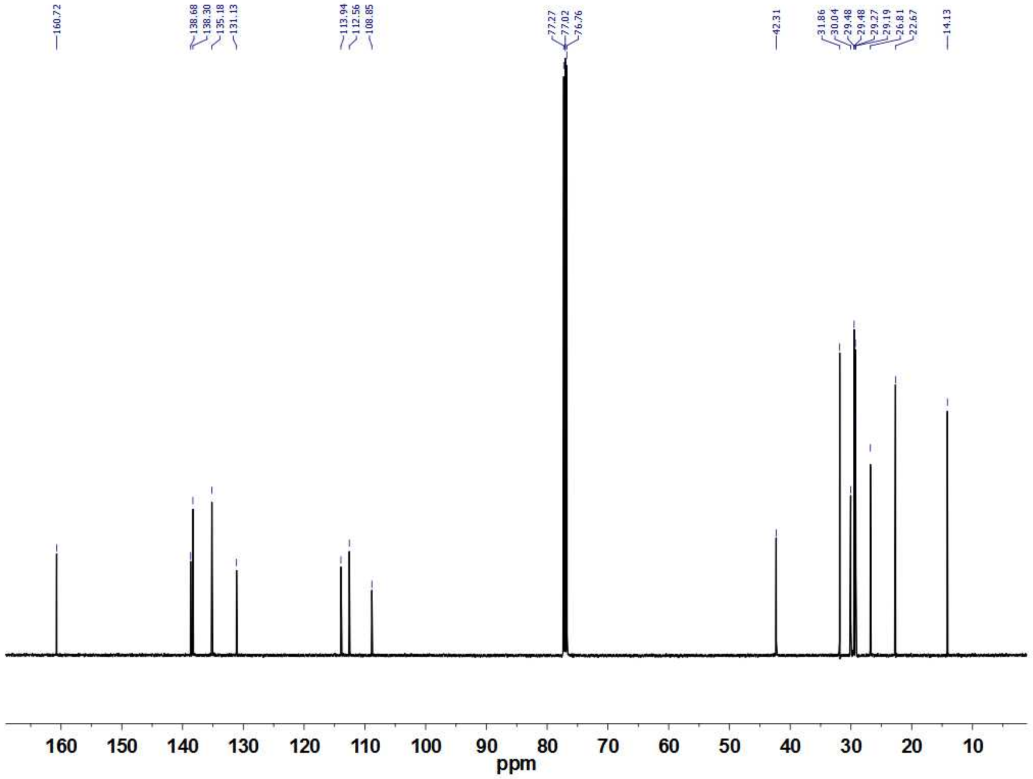

Figure S4 : ^{13}C-NMR spectrum in $CDCl_3$ of compound **M7**.

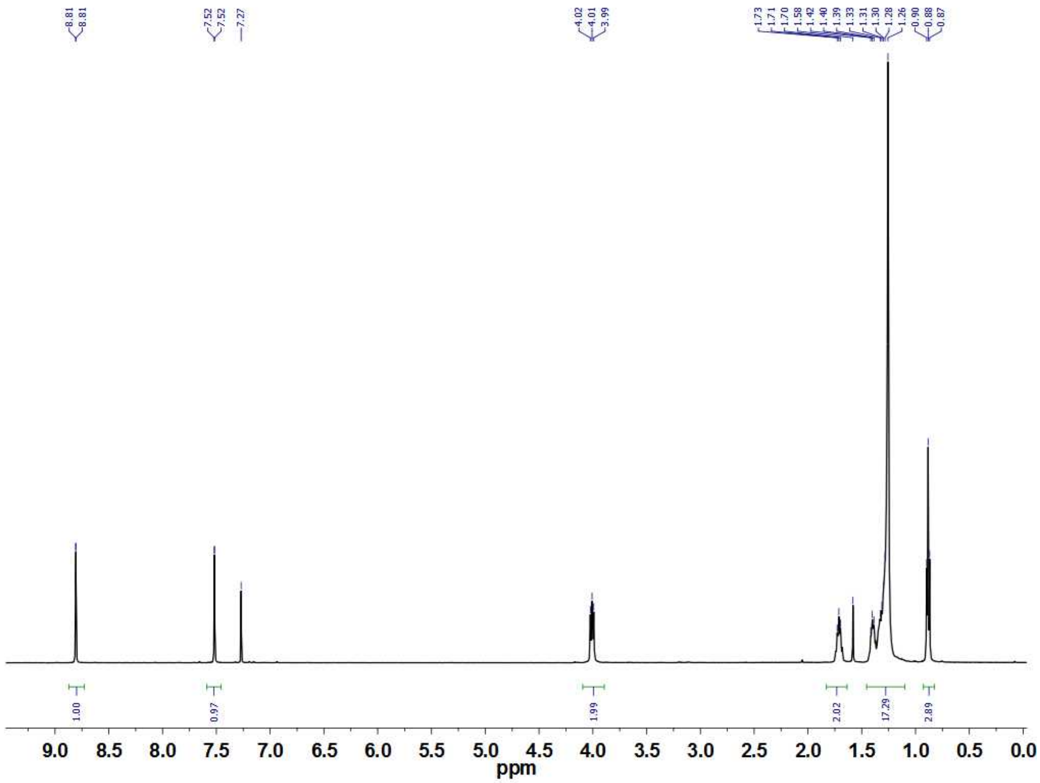

Figure S5 : ^{1}H-NMR spectrum in $CDCl_3$ of compound **4**.

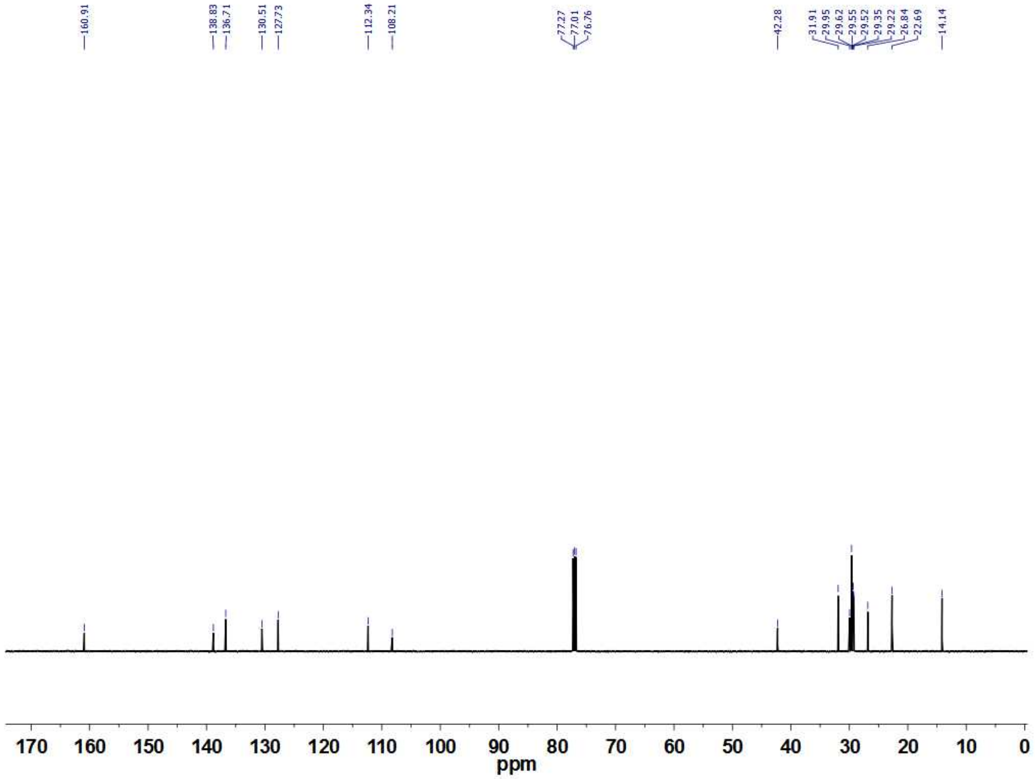

Figure S6 : ^{13}C-NMR spectrum in $CDCl_3$ of compound **4**.

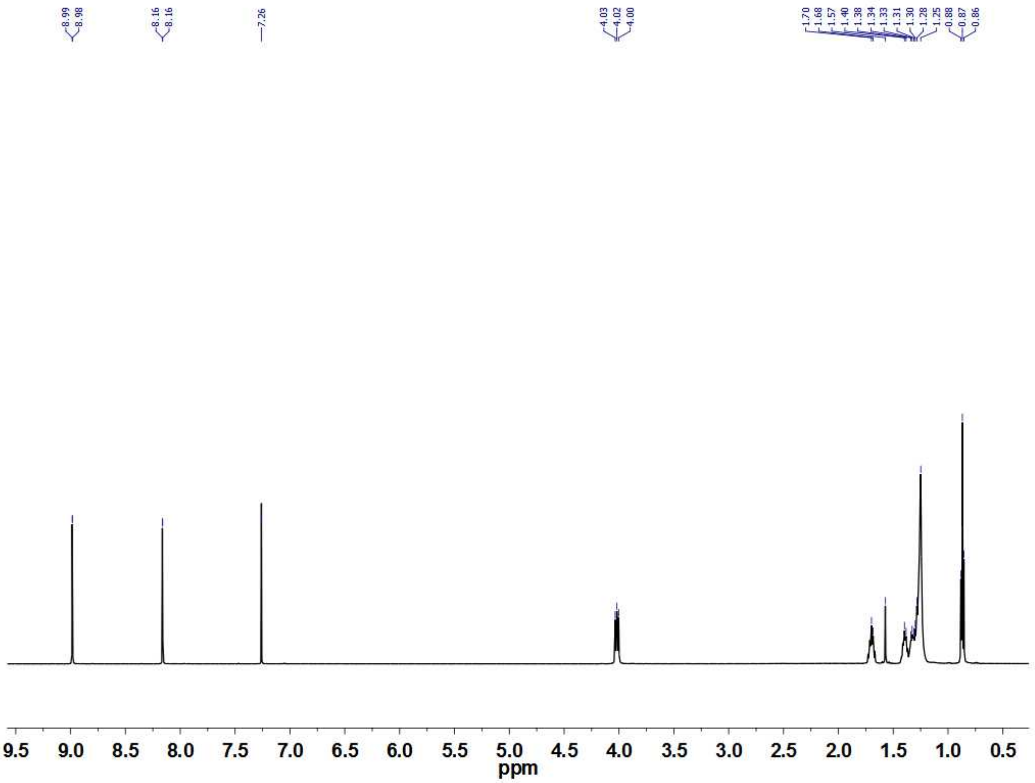

Figure S7 : ^{1}H-NMR spectrum in $CDCl_3$ of compound **M8**.

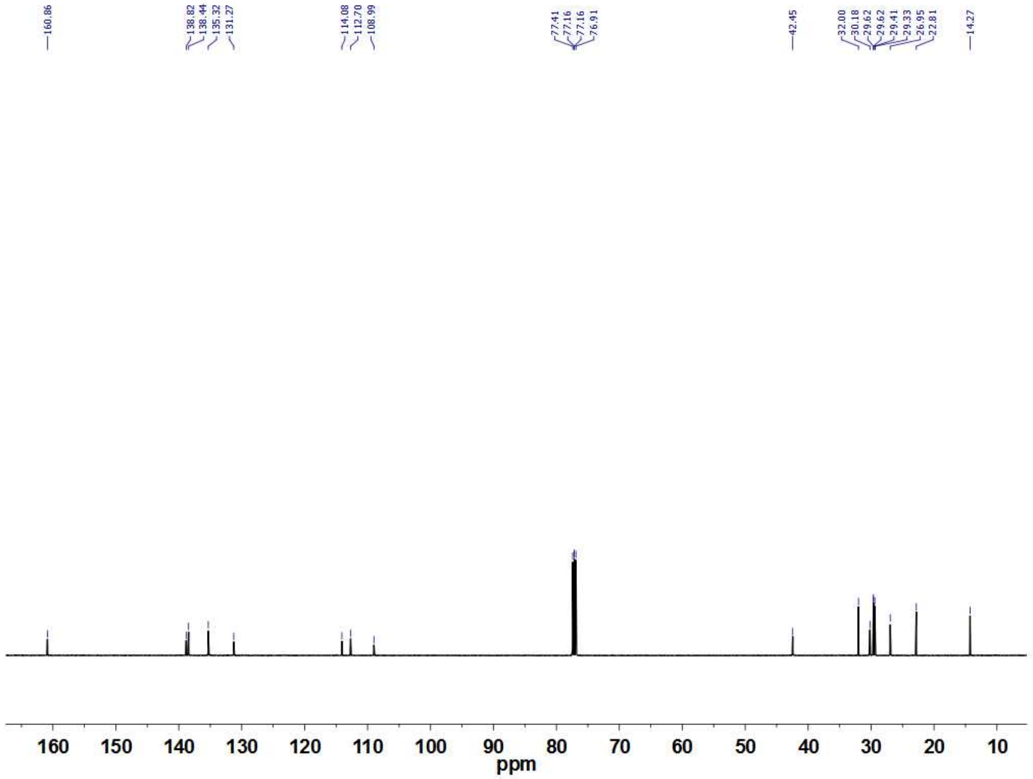

Figure S8 : ^{13}C-NMR spectrum in $CDCl_3$ of compound **M8**.

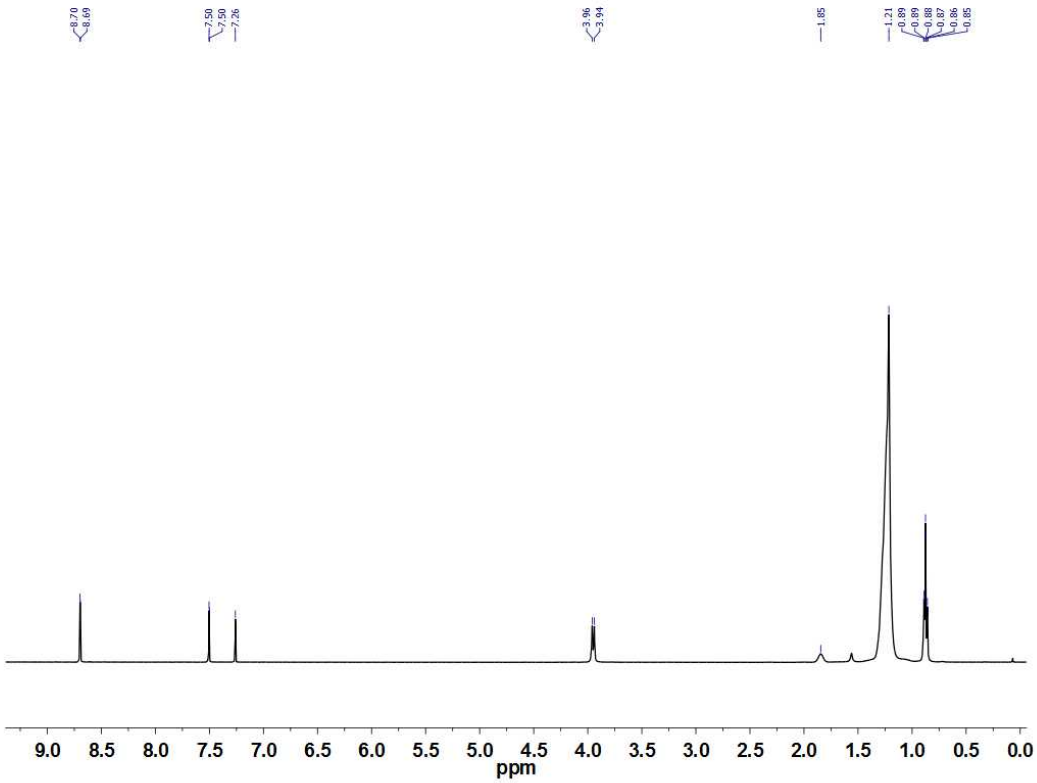

Figure S9 : ¹H-NMR spectrum in $CDCl_3$ of compound **5**.

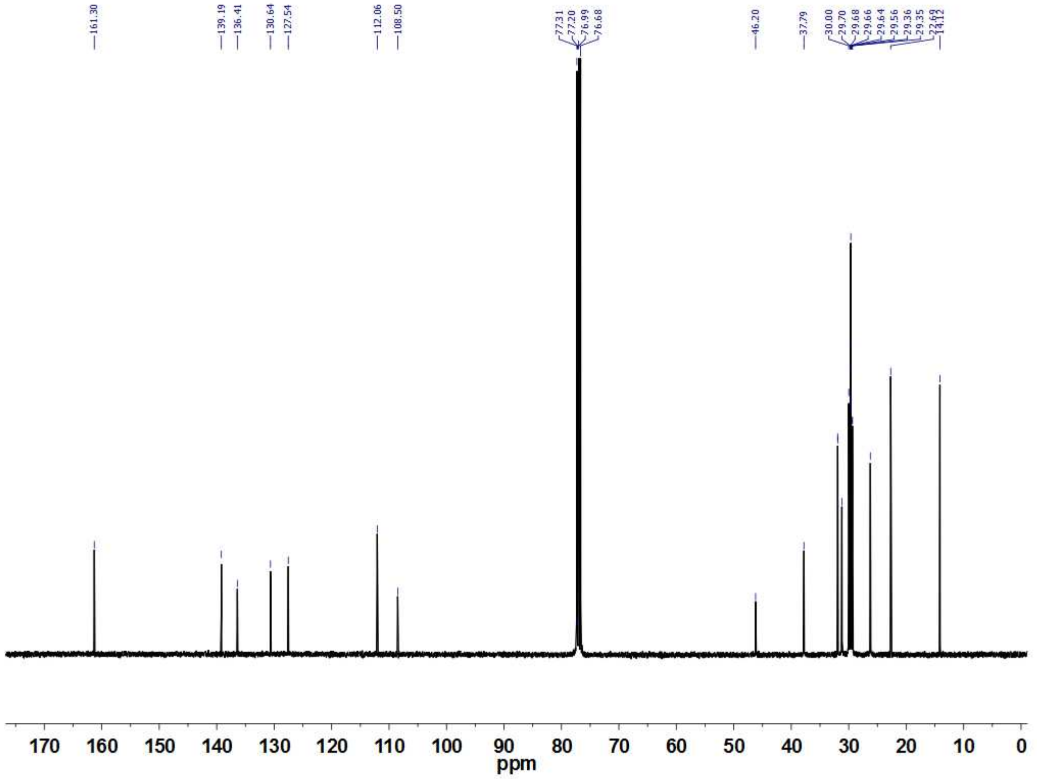

Figure S10 : ¹³C-NMR spectrum in $CDCl_3$ of compound **5**.

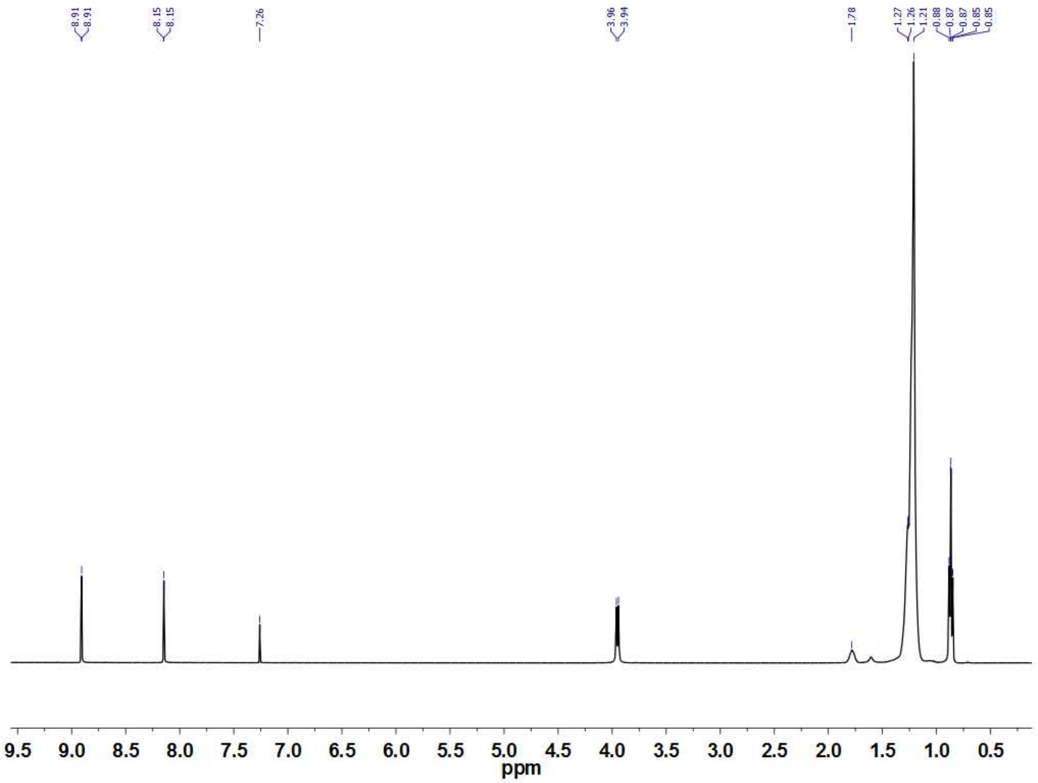

Figure S11 : ^{1}H-NMR spectrum in $CDCl_3$ of compound **M9**.

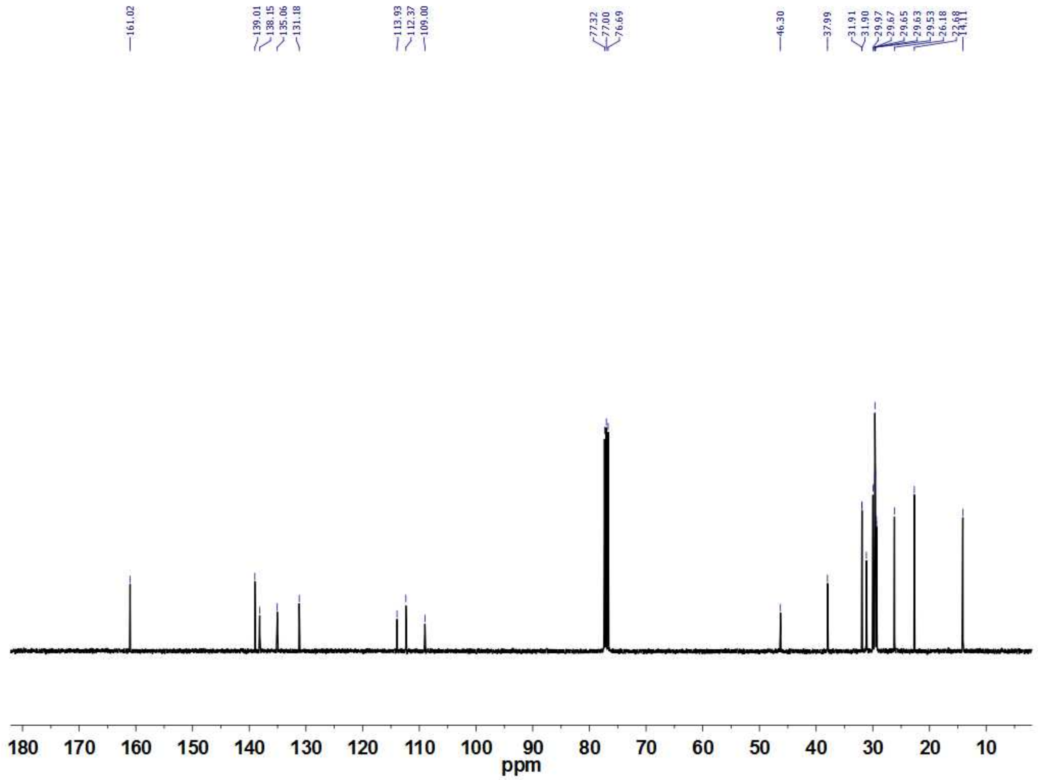

Figure S12 : ^{13}C-NMR spectrum in $CDCl_3$ of compound **M9**.

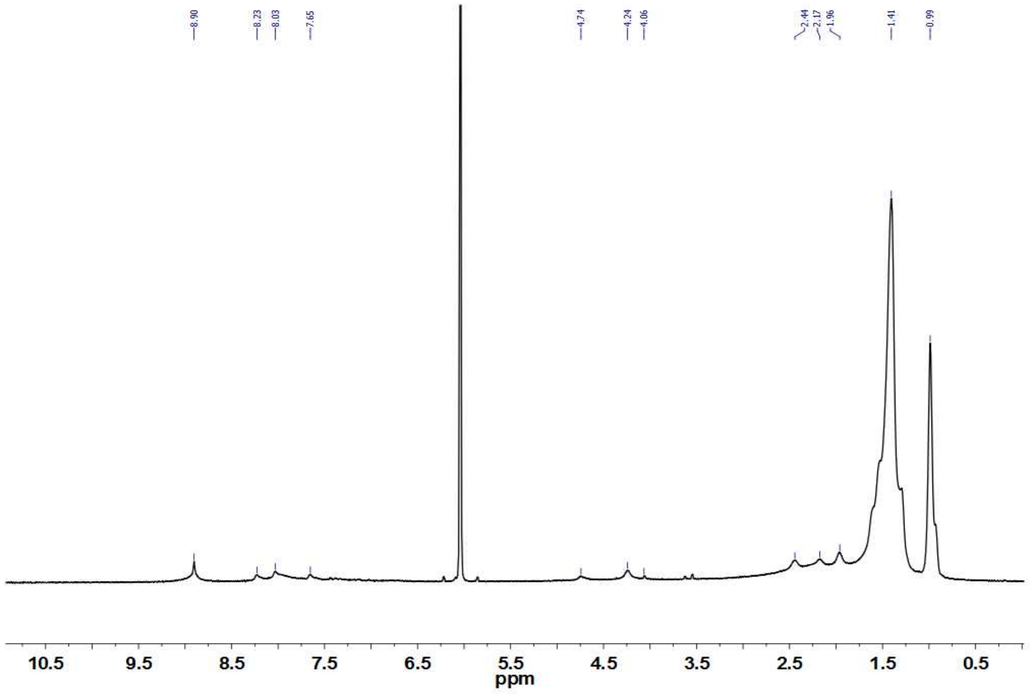

Figure S13 : ^{1}H-NMR spectrum in TCE at 110°C of polymer **P6**.

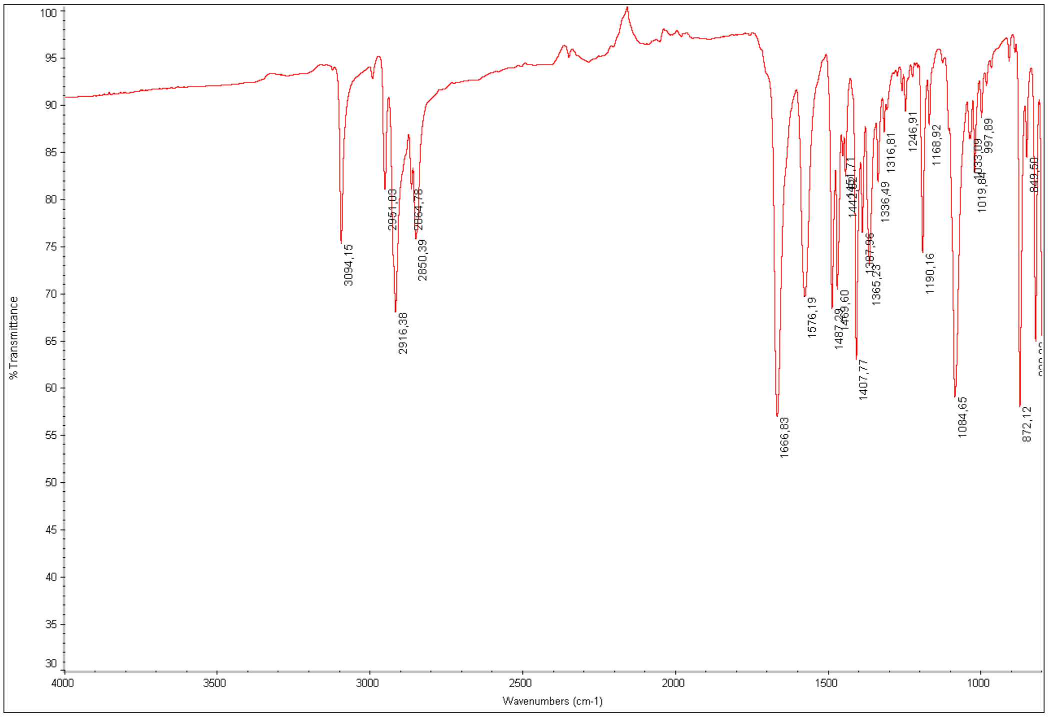

Figure S14 : Infrared spectrum of compound **3**.

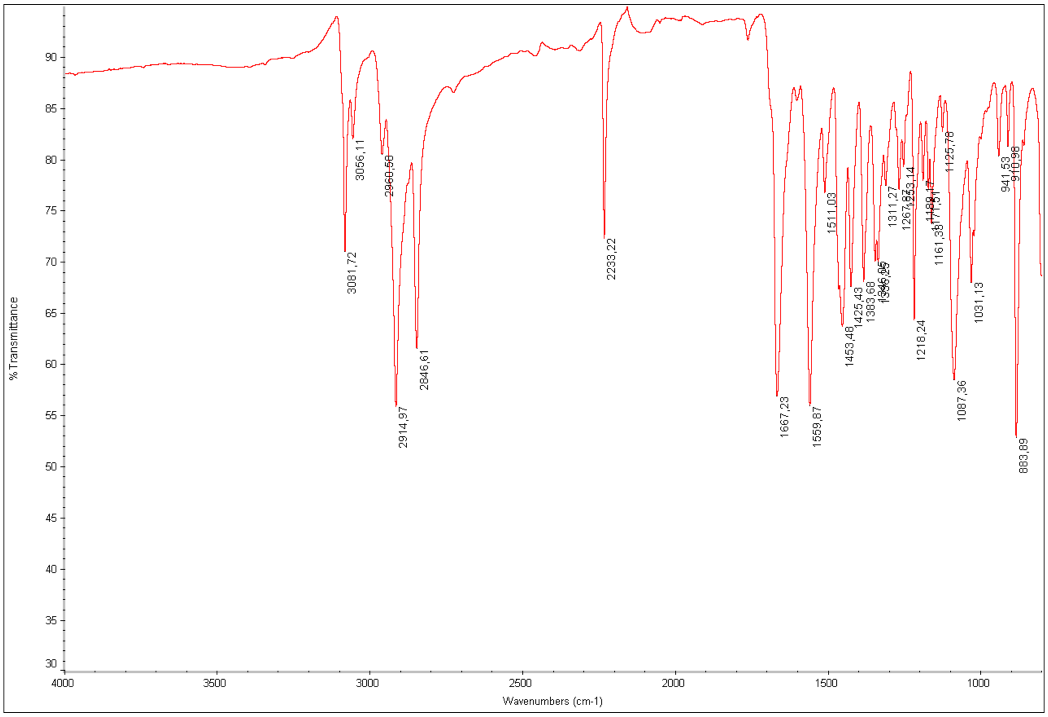

Figure S15 : Infrared spectrum of compound **M7**.

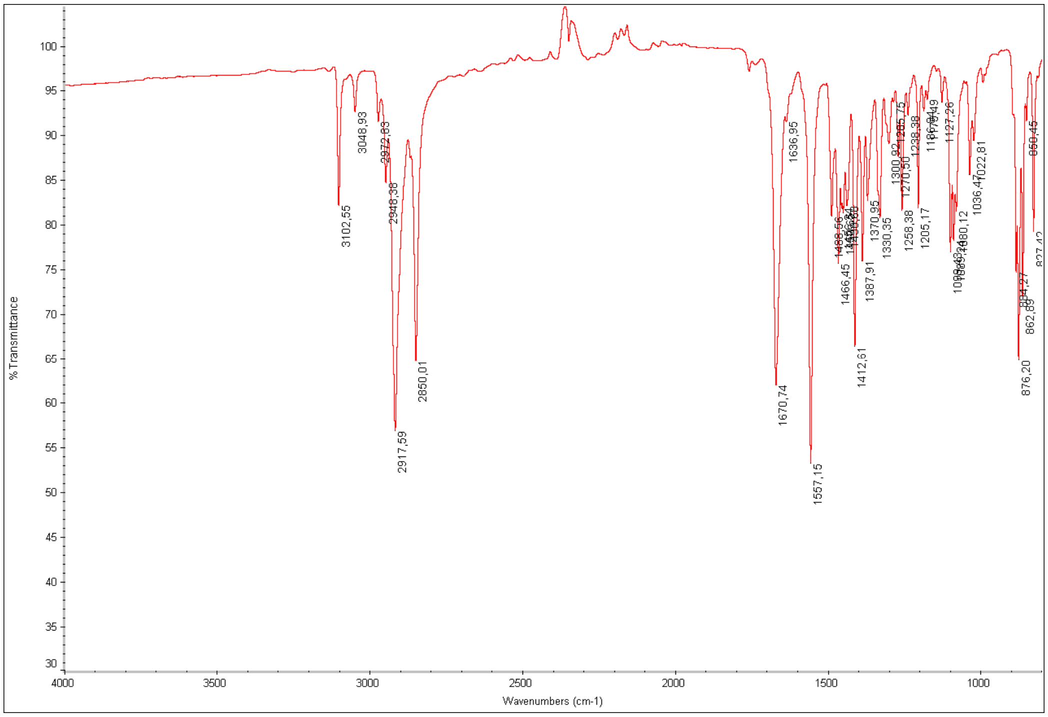

Figure 16 : Infrared spectrum of compound **4**.

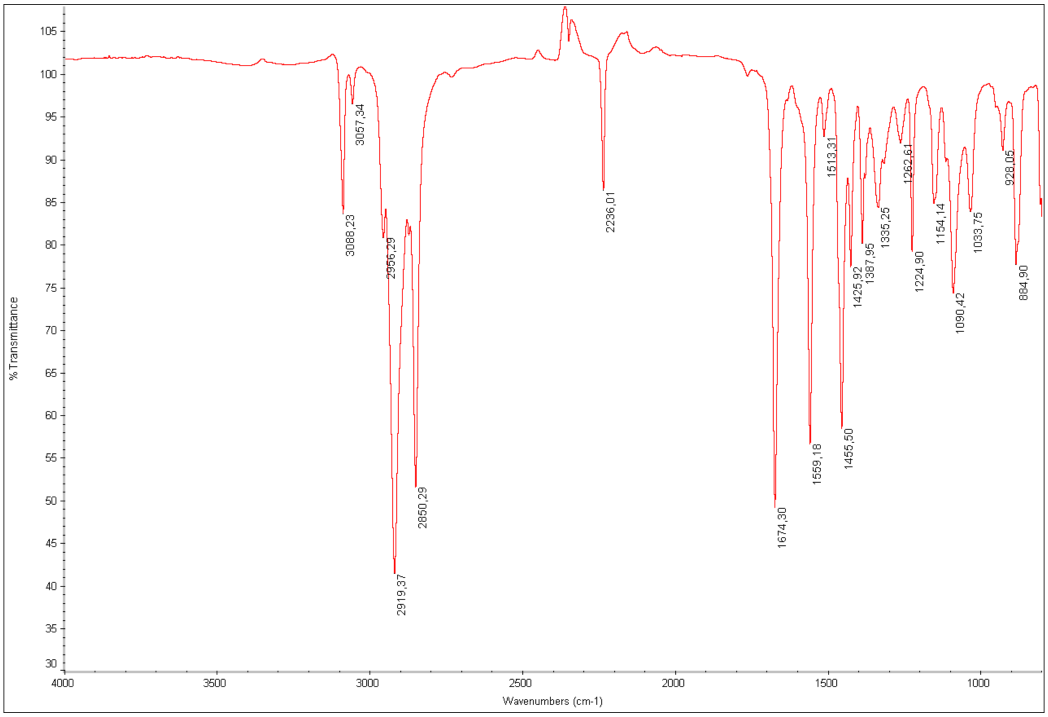

Figure S17 : Infrared spectrum of compound **M8**.

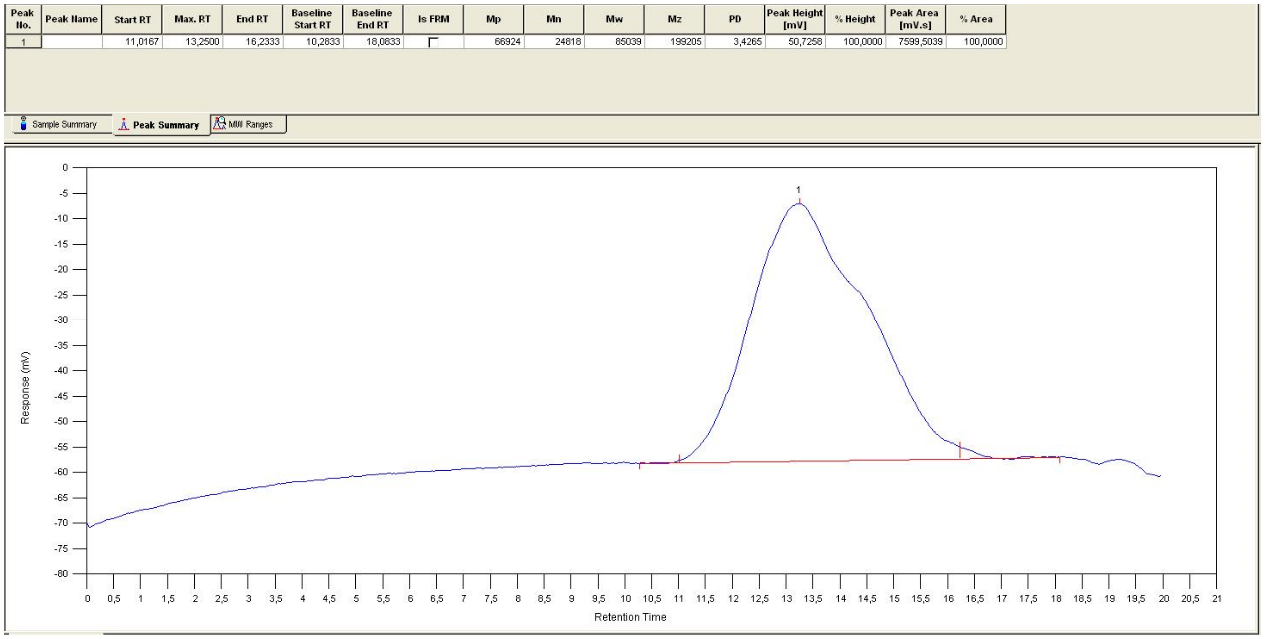

Peak No.	Peak Name	Start RT	Max. RT	End RT	Baseline Start RT	Baseline End RT	Is FRM	Mp	Mn	Mw	Mz	PD	Peak Height [mV]	% Height	Peak Area [mV.s]	% Area
1		11,0167	13,2500	16,2333	10,2833	18,0833		66924	24818	85039	199205	3,4265	50,7258	100,0000	7599,5039	100,0000

Figure S18 : GPC chromatogram for polymer **P6**

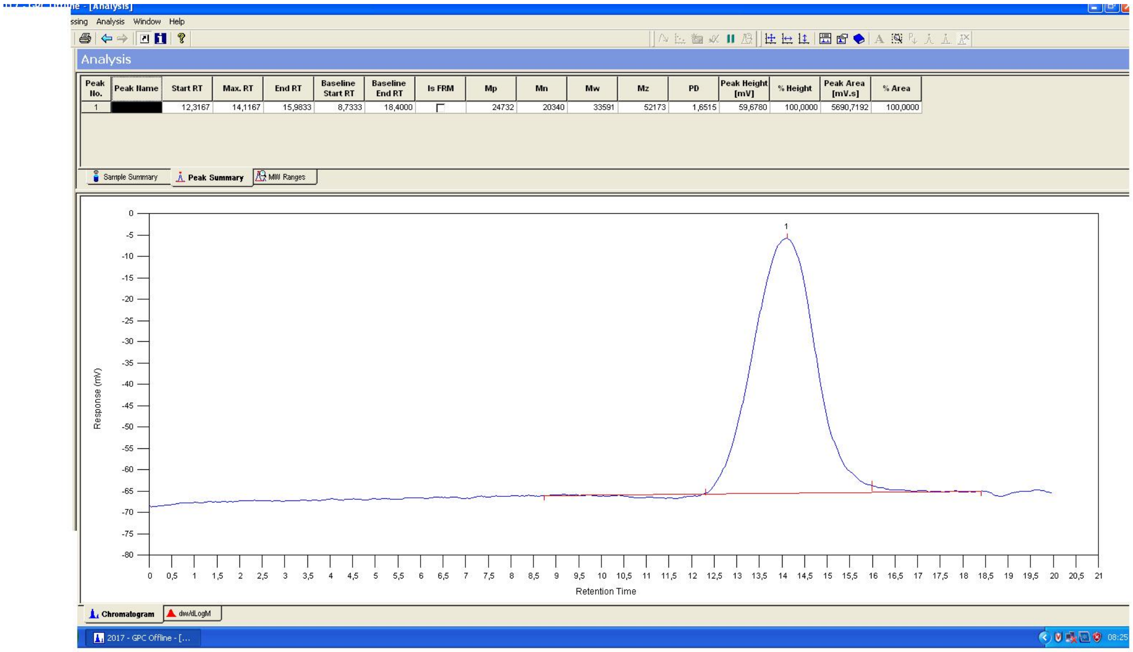

Figure S19 : GPC chromatogram for polymer **P7a**

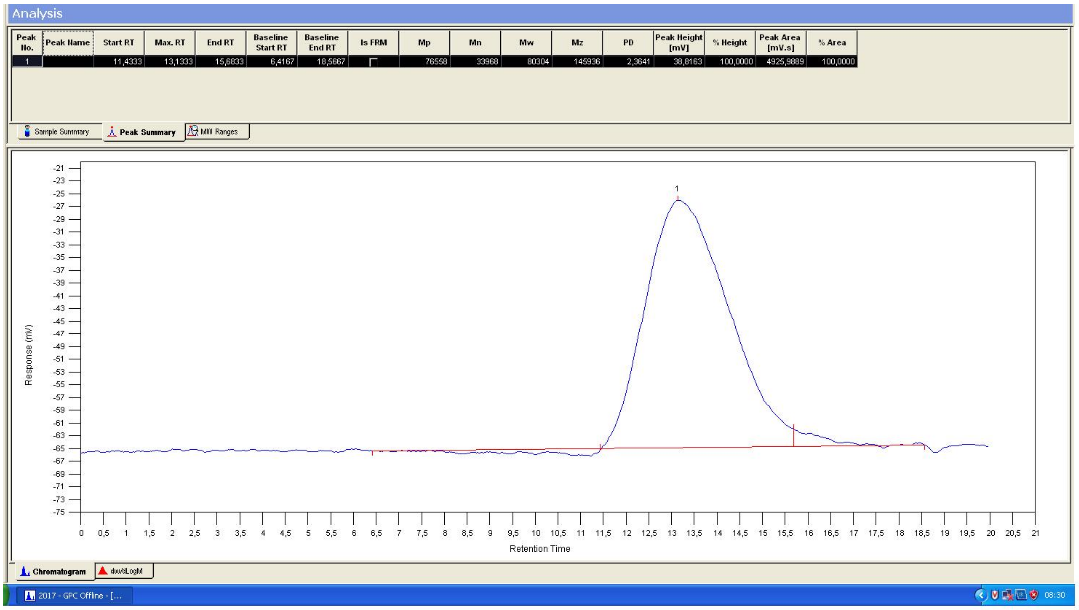

Figure S20 : GPC chromatogram for polymer **P7b**.
